# The expanding world of hybrid perovskites: materials properties and emerging applications

**DOI:** 10.1557/mrc.2015.6

**Published:** 2015-03

**Authors:** Sarah Brittman, Gede Widia Pratama Adhyaksa, Erik Christian Garnett

**Affiliations:** Center for Nanophotonics, FOM Institute AMOLF, Science Park 104, Amsterdam 1098 XG, The Netherlands

## Abstract

Hybrid inorganic–organic perovskites have emerged over the last 5 years as a promising class of materials for optoelectronic applications. Most notably, their solar cells have achieved power conversion efficiencies above 20% in an unprecedented timeframe; however, many fundamental questions still remain about these materials. This Prospective Article reviews the procedures used to deposit hybrid perovskites and describes the resulting crystallographic and morphological structures. It further details the electrical and optical properties of perovskites and then concludes by highlighting a number of potential applications and the materials challenges that must be overcome before they can be realized.

## Graphical abstract

**Figure:**
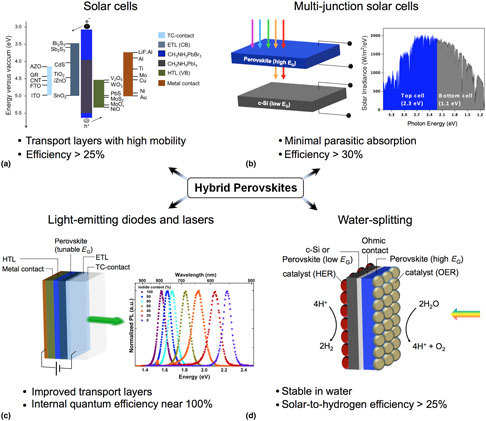

## Introduction

**Figure 1.**
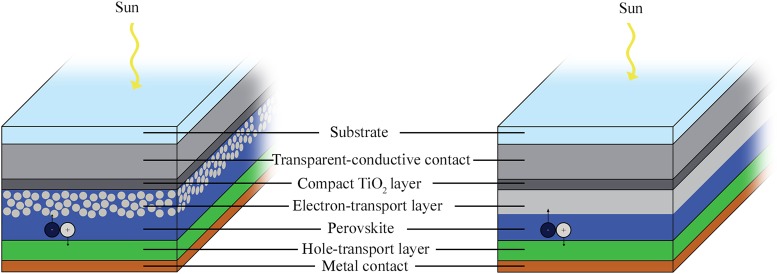
Comparison of the mesoporous (left) and planar (right) architectures used in perovskite solar cells. Most devices with high efficiency use glass/FTO/TiO_2_/CH_3_NH_3_PbI_3_/Spiro-OMeTAD/Au. Except for the glass substrate, layer thicknesses are drawn approximately to scale.

Hybrid inorganic–organic perovskites have set the materials science world abuzz because their solar cells have reached 20.1% efficiency^[^^1^^]^ after fewer than 5 years of widespread research. Perovskites began as an alternative sensitizer for dye-sensitized solar cells (DSSCs),^[^^2^^]^ but their superior charge-transport properties allowed the absorbing layer to thicken. Current mesostructured architectures still feature the perovskite absorber deposited on mesoporous TiO_2_,^[^^3^^]^ but these same excellent charge-transport properties in the perovskite also prompted study of a thin-film version of the solar cell (Fig. 1), which yields similarly high efficiencies.^[^^4^^]^

The dramatic rise of perovskites in photovoltaics has caught the attention of scientists across many fields, and the initial hype has been bolstered by investigations of perovskites’ structural, optical, and electronic properties. Perovskites offer a combination of the characteristics of inorganic and organic semiconductors: the chemical tunability of their optoelectronic properties and low-temperature and solution-based deposition recall organics; their relatively high carrier mobility, diffusion length, and radiative lifetime more resemble those of polycrystalline semiconductors (Table I). These attractive properties have inspired dreams well beyond photovoltaics, and research in sunlight-to-fuel conversion,^[^^5^^]^ light-emitting diodes,^[^^6^^,^^7^^]^ lasers,^[^^8^^–^^10^^]^ and photodetectors^[^^11^^,^^12^^]^ is already underway.

**Table I.** Comparison between CH_3_NH_3_PbI_3_-based perovskites and other photovoltaic technologies. Perovskites have many of the advantages of polycrystalline semiconductors such as CIGS and CdTe, but they are processed from solution-like organic materials or quantum dots. Material characteristics refer to values measured for materials similar to those used in the highest performing solar cells

Photovoltaic technology	Power conversion efficiency* (%)^[^^1^^]^	Absorption coefficient (cm^−1^)†	Diffusion length (μm)‡	Carrier mobility (cm^2^ Vs)‡	Carrier lifetime	Band gap (eV)	Loss-in- potential (eV)§	External radiative efficiency (%)	Stability∥	Main element		Energy-pay-back time (years)
										Critical	Toxic	
c-Si^b–f^	25.6 ± 0.5	10^2^	100–300	10–10^3^	4 ms	1.1	0.4	1.3	>25 years	–	–	1.7–4
GaAs (thin film)^g–j^	28.8 ± 0.9	10^4^	1–5	>10^3^	50 ns¶	1.4	0.3	25	>20 years	Ga	As	2.3–5
CIGS^k–r^	21.7 ± 0.6	10^3^–10^4^	0.3–0.9	10–10^2^	250 ns	1.1	0.3	10^−1^	>8 years	In, Ga	–	0.3
CdTe^s–v^	21.0 ± 0.4	10^3^	0.4–1.6	10	20 ns	1.5	0.6	10^−3^	>4.5 years	Te	Cd	0.5–1.1
Dye-sensitized^w–y^	13.0 ± 0.5 ^y^	10^3^–10^4^	0.005–0.02	10^−2^–10	1 ns**	1.6	0.7	10^−6^	<20 months	Co	–	0.5–1.5
Organic^z–hh^	11.1 ± 0.3	10^3^–10^5^	0.005–0.01	10^−5^–10^−4^^††^	10-100 μs^††^	1.6	0.7	10^−7^	<25 days	–	–	0.2–4
Quantum dot (PbS)^ii–nn^	9.2 ± 0.2^ii^	10^2^–10^3^	0.08–0.2	10^−4^–10^−2^	30 μs	1.3	0.8	10^−4^	<6 days	–	Pb	1.51
Perovskite^oo–rr^	20.1 ± 0.4	10^3^–10^4^	0.1–1.9	2–66	270 ns	1.6	0.5	10^−2^	4–42 days	–	Pb	0.9

* Under AM 1.5, 100 mW/cm^2^; † At 300 K in the vicinity of the band edge; ‡ Of the minority carrier (c-Si, GaAs, CIGS, CdTe, perovskite) or the exciton (dye-sensitized, organic, quantum dot); § *E*_G_–*qV*_oc_ (band gap—open circuit voltage); ∥ Period at which the efficiency becomes 80% of the initial value; ¶ Corrected for photon-recycling effects; ** Of a dye in solution, not on TiO_2_; †† Of the donor–acceptor blends, not the pristine material.

^a^ [1]. ^b^ Geist, J., Migdall, A., & Baltes, H. *Appl. Opt*. **27**, 3777 (1988). ^c^ [76]. ^d^ Modanese, C. et al. *Prog. Photovolt: Res. Appl*. **21**, 1469 (2012). ^e^ Tiedje, T. et al. *IEEE Trans. Electron Dev*ices **31**, 711 (1984). ^f^ Kegel, J. et al. *Appl. Surf. Sci*. **301**, 56 (2014). ^g^ [78]. ^h^ Rey-Stolle, I. & Algora, C. *Prog. Photovolt: Res. Appl*. **11**, 249 (2003). ^i^ Friedman, D.J., Olson, J.M., & Kurtz, S. *High-efficiency III–V multijunction solar cells*. (John Wiley & Sons, 2011). ^j^ Lumb, M. P. et al. *J. Appl. Phys*. **116**, 194504 (2014). ^k^ [75]. ^l^ Chirilă, A. et al. *Nature Mater.* **12**, 1107 (2013). ^m^ Repins, I. et al. *Conference paper NREL*/CP-520-46235 (2009). ^n^ Shah, A. et al. *Science* **285**, 692 (1999). ^o^ [74]. ^p^ Metzger, W.K., Repins, I.L., & Contrearas, M.A. *Appl. Phys. Lett*. **93**, 1 (2008). ^q^ Marple, D.T.F. *Phys. Rev*. **150**, 728 (1966). ^r^ Mitchell, K., Fahrenbruch, A.L., & Bube, R.H. *Appl. Phy. Lett*. **48**, 1 (1977). ^s^ Batzner, D.I. et al. *Thin Solid Films* **387**, 151 (2001). ^t^ Kato, K. et al. *Sol. Energy. Mat. Sol. Cells*. **67**, 279 (2001). ^u^ [87]. ^v^ Ma, J. et al. *Phys. Rev. Lett.* **111**, 1 (2013). ^w^ Lindstrom, H. et al. *J. Phys. Chem*. **100**, 3084 (1996). ^x^ Gebeyehu, D. et al. *Synt. Met.* **125**, 279 (2002). ^y^ Mathew, S. et al. *Nature Chem.* **6**, 242 (2014). ^z^ Kim, Y. et al. *Nature Mater.* **5**, 197 (2006). ^aa^ Chen, C-P. et al. *J. Am. Chem. Soc*. **130**, 12828 (2008). ^bb^ Espinosa, N. et al. *Sol. Energy Mat. Sol. Cells*. **95**, 1293 (2011). ^cc^ He, Z. et al. *Nature Photon.* **6**, 591 (2012). ^dd^ Jorgensen, M. et al. *Adv. Mater.* **25**, 580 (2012). ^ee^ [88]. ^ff^ [70]. ^gg^ Roes, A.L. et al. *Prog. Photovolt: Res. Appl*. **17**, 372 (2009). ^hh^ Pivrikas, A. et al. *Prog. Photovolt: Res. Appl*. **15**, 677 (2007). ^ii^ Labelle, A. J. et al. *Nano Lett.* **15**, 1101 (2015). ^jj^ Wang, X. et al. *Nature Photon.* **5**, 480 (2011). ^kk^ Chuang, C-H. M. et al. *Nature Mater.* **13**, 796 (2014). ^ll^ [89]. ^mm^ [72]. ^nn^ Jeong, K. S. et al. *ACS Nano* **6**, 89 (2012). ^oo^ [106]. ^pp^ [134]. ^qq^ [135]. ^rr^ D’Innocenzo, et al. *Nat. Commun.* **5**, 3586 (2013).

Although rapid progress has been made, many aspects of perovskites still remain mysterious. Notable examples include the role of chloride in the formation of CH_3_NH_3_PbI_3_, the origin of the electrical hysteresis seen in many solar cells, and the underlying reason for such high open-circuit voltages and low recombination in films that in most material systems would be considered of very low quality. Also, because of their promise for large-scale applications such as photovoltaics, perovskites’ stability and mechanisms of degradation must be well characterized. In these and other remaining puzzles, there is ample room for contributions from chemists, materials scientists, and physicists working in both experiment and theory. Since perovskite solar cells developed from DSSCs and the materials themselves exhibit properties in common with both organic absorbers and polycrystalline semiconductors, the knowledge gained from these fields is invaluable in advancing current understanding.

While many have chronicled the development of perovskite photovoltaics,^[^^13^^–^^16^^]^ this Prospective Article focuses on the properties of hybrid perovskites themselves. It begins with their structure and methods of deposition and then surveys their unique electrical and optical properties, noting areas that still require further investigation. The remainder of this article is devoted to emerging applications of perovskites beyond single-junction photovoltaics along with a discussion of some challenges that must be tackled if these materials are to make a technological impact. Although much has been learned from the frenzy of recent research efforts, exciting work in developing new hybrid perovskites and in understanding existing materials still remains.

## Crystal structures

**Figure 2.**
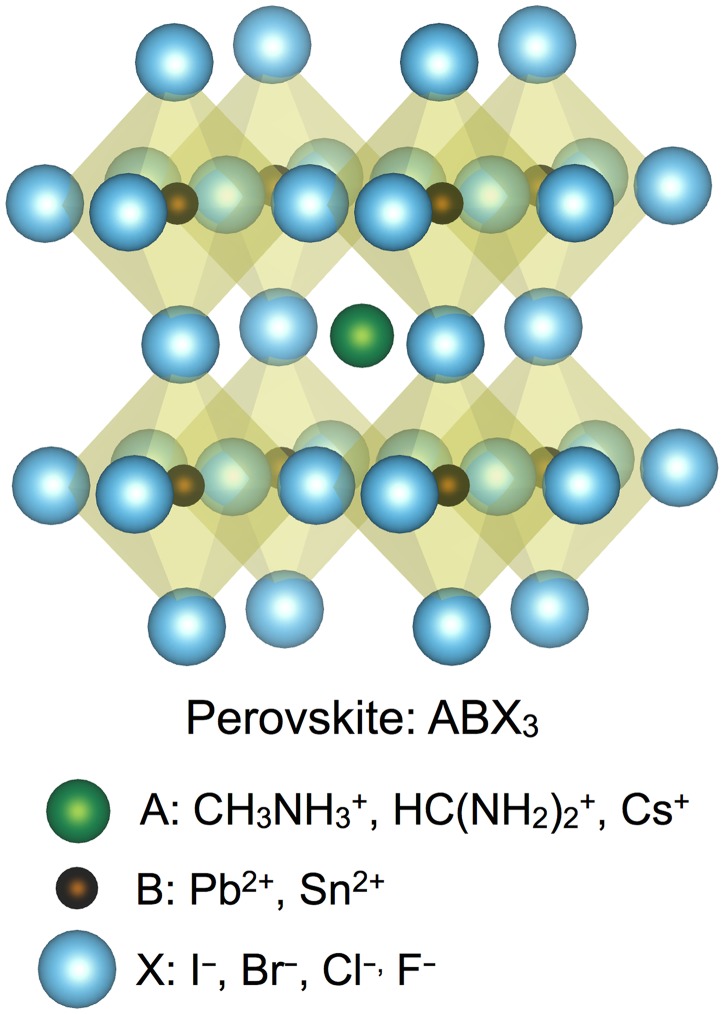
The perovskite crystal structure with the general form AB*X*_3_, in which A^+^ is confined within a cage determined by the octahedral coordination of B^2+^ with X^−^ anions. The sizes of the spheres are given by the ionic radii of CH_3_NH_3_^+^, Pb^2+^, and I^−^.^[^^13^^,^^17^^]^ The most common cations and anions that have been used in each position within the 3D halide perovskites are listed. The wider variety of materials that have been used in 2D halide hybrid perovskites can be found in the review by Mitzi.^[^^31^^]^

The general, formula of the perovskite crystal structure is AB*X*_3_, in which A is the larger cation, B is the smaller cation, and *X* is the anion (Fig. 2). In the most common hybrid perovskites these are, respectively, CH_3_NH_3_^+^, Pb^2+^, and a halide (I^−^, Br^−^, or Cl^−^) or mixture of halides. The smaller *B* cation is octahedrally coordinated by *X* anions, with the octahedra sharing corners in a three-dimensional (3D) lattice. The larger *A* cations fill the vacancies between the octahedra and have twelve *X* nearest neighbors. The possibilities for cations A and B are limited by the stability of the resulting structure, which can be estimated geometrically by the Goldschmidt tolerance factor and an octahedral factor introduced by Li.^[^^17^^]^ Although these two parameters are quite successful in predicting the formation of a perovskite, predicting which distortions occur to the archetypical cubic structure is more difficult because these geometric factors do not account for ionic or covalent-bonding interactions, vibrational motion, or hydrogen bonding. These distortions reduce the symmetry of the lattice into a tetragonal or orthorhombic space group, but the distorted perovskite retains the chemical formula and coordination numbers of the archetypical cubic structure.

To understand the possible crystal symmetries of the CH_3_NH_3_Pb*X*_3_ perovskites, where *X* is I^−^, Br^−^, or Cl^−^, it is instructive to begin from the cubic structure (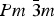) that exists at high temperatures and to consider the changes that occur upon cooling. All three perovskites transition from the cubic phase through one or more tetragonal phases and then to an orthorhombic phase at low temperature. CH_3_NH_3_PbI_3_ exists at room temperature in its tetragonal phase (*I*4/*mcm*), in which the CH_3_NH_3_^+^ cations rotate freely as determined by neutron diffraction,^[^^18^^]^ infrared (IR) spectroscopy,^[^^19^^]^ and nuclear magnetic resonance.^[^^20^^]^ This rotational freedom imparts pseudo-spherical symmetry to the CH_3_NH_3_^+^, which is necessary for a cubic structure. When CH_3_NH_3_PbI_3_ transitions to its orthorhombic phase, the CH_3_NH_3_^+^ cations become oriented. In contrast, CH_3_NH_3_PbCl_3_ exists in its cubic phase at room temperature, and its CH_3_NH_3_^+^ cations show ordering at the cubic–tetragonal transition.^[^^19^^,^^20^^]^ Lastly, the CH_3_NH_3_PbBr_3_ perovskite exists at room temperature in its cubic phase like CH_3_NH_3_PbCl_3_. It passes through one tetragonal phase that corresponds to that of the chloride and then through a second tetragonal phase that corresponds to that of the iodide.^[^^21^^]^ The ordering of the CH_3_NH_3_^+^ cations occurs at the tetragonal–orthorhombic transition, as is the case for the iodide. Because the ordering and movement of the CH_3_NH_3_^+^ cations are hypothesized to be linked to the hysteresis seen in the electrical transport of the perovskites (discussed in Section 5.3), these structural transitions induced by temperature provide insight into changes that could be induced by applied voltage.

To determine the structure and space group of perovskites experimentally, x-ray diffraction (XRD) is typically used, but it provides limited information. For example, in hybrid halide perovskites such as CH_3_NH_3_PbI_3_, the position of the CH_3_NH_3_^+^ is determined only indirectly by its effect on the inorganic portion of the crystal. Analyzing the position and orientation of this molecule within the 3D framework, particularly the orientation of the C–N axis, is important since alignment of the C–N dipoles is proposed to be the source of ferroelectricity in CH_3_NH_3_PbI_3_ (discussed in Section 5.3). As a complementary technique to XRD, neutron diffraction is much more sensitive to lighter elements. It can be used to determine the location of the C and N atoms within the lattice for phases with ordered cations and to provide some insight into favored orientations for phases with disordered cations.^[^^18^^]^ It is noteworthy that neutron diffraction has been used to reassign the space group for the orthorhombic phase of CH_3_NH_3_PbBr_3_,^[^^18^^]^ which suggests that the assignments proposed earlier^[^^21^^]^ would benefit from confirmation. Also, transmission electron microscopy and selected-area electron diffraction can be applied to analyze the local crystal structure and grain distribution.^[^^22^^,^^23^^]^

## Methods of deposition

**Figure 3.**
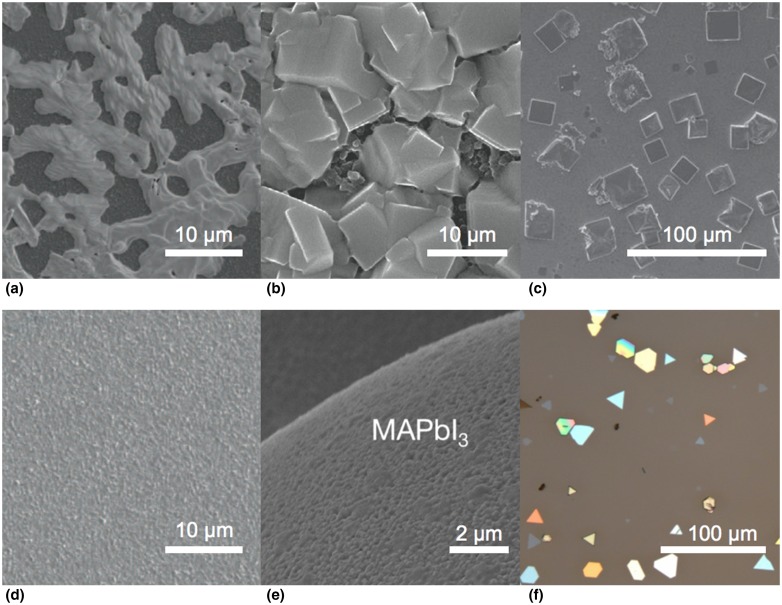
A gallery of morphologies of the hybrid perovskites. SEM images of (a) solution-deposited CH_3_NH_3_PbI_3–*x*_Cl_*x*_, (b) CH_3_NH_3_PbI_3_ deposited by the two-step solution method, (c) crystals of CH_3_NH_3_PbBr_3_ deposited from DMF solution, (d) CH_3_NH_3_PbI_3–*x*_Cl_*x*_ deposited by co-evaporation of PbCl_2_ and CH_3_NH_3_I in vacuum, and (e) a microsphere conformally coated with CH_3_NH_3_PbI_3_ produced by the conversion of PbS deposited by atomic-layer deposition. (f) An optical image of CH_3_NH_3_PbI_3_ platelets synthesized by the two-step process in vapor phase. Panels (a) and (d) are reproduced with permission from Ref. 4. Copyright 2013, Nature Publishing Group. Panel (b) is reproduced with permission from Ref. 36. Copyright 2014, Nature Publishing Group. Panel (c) is reproduced with permission from Ref. 34. Copyright 2014, Wiley-VCH Verlag GmbH & Co. Panel (e) is reproduced with permission from Ref. 9. Copyright 2014, Wiley-VCH Verlag GmbH & Co. Panel (f) is reproduced with permission from Ref. 10. Copyright 2014, American Chemical Society.

One of the key attractions of perovskites is the apparent simplicity of their preparation; however, often a simple procedure belies complex chemistry and dynamics that give the material its unique morphology and properties (Fig. 3). As discussed below, the formation of CH_3_NH_3_PbI_3_ in the presence of chloride anions—termed perhaps misleadingly CH_3_NH_3_PbI_3−*x*_Cl_*x*_ in much of the literature—is an example of such a case.

Lead-halide perovskites have been produced primarily by two methods: precipitation from solution and deposition from the vapor phase. Additionally, two-step processes in both solution and vapor phases have been explored. In these reactions, the lead halide is prepared first and then exposed to the methylammonium halide to yield the perovskite. A few solid-state reactions have been reported^[^^24^^]^ as well as a multi-step synthesis based on the conversion of PbS deposited by atomic-layer deposition to PbI_2_.^[^^25^^]^

### Solution-based methods

The simplest method for depositing perovskites is precipitation from solution: the precursor metal halide and organic halide are dissolved, and evaporation of the solvent by heating or spincoating yields perovskite crystals. Common solvents include Lewis bases such as dimethylformamide (DMF), dimethylsulfoxide (DMSO), and gamma-butyrolactone. Little is known about the species present in solution, although solvents such as DMSO and DMF are known to coordinate to Pb^2+^ halide salts,^[^^26^^–^^28^^]^ and Pb^2+^ halide complexes are common in aqueous solutions of excess halide.^[^^29^^,^^30^^]^ The morphology of the resulting crystals and films produced from solution depends critically on the choice of solvent(s), the mode of their removal, and also on the substrate's morphology and surface chemistry.^[^^31^^–^^36^^]^ For example, using DMSO in a mixture of solvents produces an intermediate phase after spincoating that then crystallizes into the perovskite upon annealing.^[^^33^^]^ Forming continuous, pinhole-free films has proven to be challenging and remains a chief concern in fabricating solar cells of high efficiency.^[^^14^^]^ Addition of a hydrohalic acid to the precursor solution has offered some improvement in the film morphology, but the underlying reason remains unclear.^[^^34^^,^^37^^]^

The single-step solution process has also been the method of choice for creating controllable mixtures of perovskites with higher band gaps than CH_3_NH_3_PbI_3_ such as I–Br perovskites^[^^38^^–^^40^^]^ and Br–Cl perovskites.^[^^39^^,^^41^^]^ In these cases, the global stoichiometry of the resulting films appears to follow the stoichiometry of the precursors in solution, although chemical homogeneity on the nanoscale has not yet been confirmed.

In order to gain better control over the deposition of CH_3_NH_3_PbI_3_, a two-step solution process was developed and has in general produced solar cells with higher efficiencies than the single-step process,^[^^42^^]^ which could be due to the film's different carrier type and doping concentration (discussed in Section 5.1). In this approach, either PbI_2_ or PbCl_2_ is dissolved in DMF, spincoated onto a substrate, and then dried into a film. This film is then either dipped into an alcoholic solution of excess CH_3_NH_3_I alone or a CH_3_NH_3_I–CH_3_NH_3_Cl mixture^[^^42^^,^^43^^]^ or spincoated repeatedly with such a solution to convert the lead-halide film into the pervoskite.^[^^44^^]^ When such a process is applied to PbCl_2_, CH_3_NH_3_PbI_3_ is formed, presumably because according to the theory of hard and soft acids and bases^[^^45^^,^^46^^]^ the softer, more polarizable I^−^ has much greater affinity for Pb^2+^ than the much harder Cl^−^.^[^^47^^]^ As this approach requires diffusion of the CH_3_NH_3_^+^ cations into the lead-halide matrix, it is possible that there is a thickness limitation on the films formed by this approach or higher temperatures or longer times are required to produce thicker films.^[^^42^^]^

### Vapor-phase methods

Vapor-phase methods have also been used to produce hybrid perovskites, and their morphology and crystal structure differ from perovskites produced from solution. Co-evaporation of PbCl_2_ and CH_3_NH_3_I in a vacuum deposition chamber yields highly uniform CH_3_NH_3_PbI_3_ films with excellent photovoltaic characteristics.^[^^4^^]^ In situ XRD^[^^48^^]^ has been used to track the phase of the perovskite formed in a similar reaction and indicates that a large miscibility gap exists between CH_3_NH_3_PbI_3_ and CH_3_NH_3_PbCl_3_. The evaporation rates of the precursors determine the composition of the resulting perovskite, and the CH_3_NH_3_PbI_3_ produced was confirmed to be cubic rather than the usual tetragonal phase that exists at room temperature.

Two-step processes involving vapor-phase reactions, similar to those performed in solution, have also been demonstrated. In the vapor-assisted solution process, PbI_2_ is deposited from solution and then converted to the perovskite by exposure to CH_3_NH_3_I vapor.^[^^49^^]^ Platelets of lead halides have also been synthesized directly from the evaporation of lead-halide powders and then converted to perovskite platelets by exposure to methylammonium halide vapor.^[^^50^^]^ This two-step, vapor-phase method yields pure iodide, bromide, and chloride perovskites, as well as some mixtures. Interestingly, an I–Cl mixture was produced that exhibited blue-shifted photoluminescence (PL) (~750 nm) as compared with CH_3_NH_3_PbI_3_ (~760 nm), suggesting that the miscibility gap between the two materials might be relaxed in these platelets, whose thicknesses range between 60 and 260 nm.

### Role of Cl^−^ in the deposition of CH_3_NH_3_PbI_3_

The earliest perovskite solar cells were produced from CH_3_NH_3_PbI_3_, but it was later discovered that incorporating Cl^−^ during the crystallization process dramatically improved their photovoltaic performance.^[^^51^^]^ Subsequent studies confirmed the superior properties of CH_3_NH_3_PbI_3−*x*_Cl_*x*_: its optical absorption is stronger near the band gap,^[^^52^^–^^54^^]^ it exhibits longer PL lifetimes,^[^^55^^]^ and it yields nearly equal electron and hole diffusion lengths of ~1 μm,^[^^56^^]^ as compared with the ~100–300 nm measured for typical CH_3_NH_3_PbI_3_.^[^^57^^]^

At first it was proposed that incorporation of Cl^−^ into the film to form CH_3_NH_3_PbI_3−*x*_Cl_*x*_ was responsible for this enhancement; however, mounting evidence indicates that Cl^−^ might not be present in the bulk of the CH_3_NH_3_PbI_3_ films synthesized with Cl^−^. From XRD, CH_3_NH_3_PbI_3−*x*_Cl_*x*_ has identical structure to CH_3_NH_3_PbI_3_. In films deposited on planar substrates, no Cl^−^ has been detected via energy-dispersive x-ray spectroscopy (EDS), electron-energy loss spectroscopy or x-ray photoelectron spectroscopy (XPS).^[^^43^^,^^52^^,^^58^^]^ The detection limit of the EDS measurement was reported as 0.1 wt%, corresponding to 2 at.%,^[^^52^^]^ and similar detection limits are typical for the other techniques; consequently, none of these techniques rules out incorporation at doping levels, which is still sufficient to play a key role in the passivation of defects. Examples of important trace elements in other photovoltaic technologies include chloride within cadmium telluride (CdTe) and sodium within CuIn_*x*_Ga_1−*x*_Se_2_ (CIGS).^[^^59^^]^ In these cases, the impurities are found segregated near the surfaces and grain boundaries, so a similar process of passivation might be at work in the perovskites. Additionally, a recent study using depth-sensitive, angle-resolved XPS on ultrathin films deposited on compact TiO_2_ suggests that the Cl^−^ is localized to the TiO_2_–perovskite interface,^[^^54^^]^ which could explain why Cl^−^ was detected via EDS in the perovskite deposited on mesoporous Al_2_O_3_.^[^^52^^]^ If it is indeed localized to the interface, then the high surface area of meso-structured templates can enhance the signal above the limit of detection.

Several hypotheses have been proposed to explain the improved performance of CH_3_NH_3_PbI_3−*x*_Cl_*x*_. Since the film's morphology changes from its Cl-free counterpart, one proposal is that an insoluble lead-chloride compound acts as a nucleation site for the iodide perovskite, enhancing its nucleation rate and density; this change in morphology determines the film's properties.^[^^22^^,^^60^^,^^61^^]^ The location of the Cl^−^ at the perovskite-scaffold interface is consistent with this hypothesis. Other work suggests that growing the film in a CH_3_NH_3_^+^-rich or I^−^-poor environment changes the defect structure of the film, which improves the performance.^[^^61^^,^^62^^]^

## Tuning the band gap with composition

Perhaps the best-known characteristic of hybrid perovskites is that their band gap can be tuned widely from the blue to the red spectral regions. Such range is accomplished by replacing the halide. The valence and conduction bands of perovskites are determined by the inorganic octahedron. The valence band is composed of hybridized Pb 6*s* and halide *p* orbitals, while the conduction band is primarily Pb 6*p* in character with minor contributions from the halide *s* orbitals.^[^^63^^–^^67^^]^ The organic cation does not contribute to these bands but modulates the band gap by modifying the lead-halide bond distance;^[^^31^^,^^37^^]^; consequently, the shifts produced by substituting the organic cation are less pronounced than those produced by substitution of the halide.^[^^37^^]^ The full tunability of the band gap for CH_3_NH_3_Pb*X*_3_ has been demonstrated for I–Br compositions^[^^38^^,^^40^^]^ and strongly suggested for Br–Cl compositions^[^^39^^,^^41^^]^ from UV–visible absorption spectroscopy and PL measurements. Changing the I–Cl ratio does not yield a change in band gap because of their much larger difference in ionic size.^[^^54^^]^ Instead, phase segregation results.^[^^48^^]^

As has been seen in the debate surrounding CH_3_NH_3_PbI_3−*x*_Cl_*x*_, confirmation of the uniformity and stoichiometric composition of perovskite mixtures is a difficult task. XRD peak positions and broadening provide a large-scale account of the crystalline portions of composite films and have been used as evidence of homogeneous mixtures. Unless performed at a synchrotron with high spatial resolution, however, XRD does not tell the full story of amorphous inclusions or microscopic inhomogeneity. Element-sensitive techniques such as XPS and EDS contribute further insight, yet XPS is sensitive only to the surface and cannot probe the hundreds-of-nanometer-thick films used in photovoltaic cells without depth profiling via sputtering. EDS has already proven useful, although overlapping peaks between lead and chloride^[^^52^^]^ and concerns regarding damage to the hybrid perovskites from the electron beam make it less helpful for these materials than for inorganic semiconductors. On the other hand, secondary-ion mass spectrometry is an alternative that has yet to be applied to hybrid perovskites. Also, spatially resolved optical techniques such as mapping using Raman spectroscopy and PL spectroscopy have the potential to yield compositional information on the sub-micrometer length scale. Such detailed information is essential to uncovering the relationships between the composition and properties of films of hybrid perovskites, in particular for the compositions with higher band gaps that are interesting for tandem solar cells, light-emitting diodes, and conversion of sunlight into chemical fuels.

## Electrical properties

For solution-processed semiconductors with grain sizes below a few micrometers, hybrid perovskites exhibit unprecedented carrier transport properties that enable their stellar performance in photovoltaics. Quantitatively characterizing this transport, understanding the materials properties that give rise to it, and developing ways to improve it are all key directions for research. Additionally, perovskite solar cells are also known for their hysteresis, which still remains unexplained.

### Intrinsic electrical properties

The starting point for characterizing materials electrically is always quantitative determination of properties such as carrier type, concentration, and mobility; however, in the case of perovskites, these properties have exhibited a large range of values often influenced by the method used to prepare the perovskite. Additionally, the lack of smooth and uniform films on which to perform measurements can make the determination of intrinsic electrical properties challenging since conventional techniques often assume the sample exhibits a specific geometry.

Thermoelectric measurements of the Seebeck coefficient, Hall measurements of the conductivity's response to an applied magnetic field, or a thin-film transistor's response to a gating electric field are typical methods used to determine the carrier type. Early resistivity, Seebeck, and Hall measurements on polycrystalline CH_3_NH_3_PbI_3_ indicated *n*-type conductivity, a carrier concentration of ~10^9^ cm^−3^, and an electron mobility of 66 cm^2^/V/s.^[^^24^^]^ A more detailed study determined that tuning the stoichiometry of the precursors during solution-phase synthesis can adjust the carrier concentration and even switch the carrier type to *p*-type when excess CH_3_NH_3_I is used, which is the case in two-step syntheses; it is proposed that iodide vacancies are responsible for the *n*-type conductivity, and electron concentrations of ~10^17^–10^18^ cm^−3^ were measured.^[^^68^^]^ From Hall measurements, the electron mobility for *n*-type films deposited from stoichiometric precursors was determined to be 3.9 cm^2^/V/s,^[^^68^^]^ which is in accord with the 8 cm^2^/V/s measured using terahertz spectroscopy,^[^^55^^]^ a technique that usually provides an upper limit because it neglects long-range carrier scattering^[^^68^^]^; however, it is not surprising that the mobility depends strongly on the preparation of the film. In the most extreme case, for instance, CH_3_NH_3_SnI_3_ prepared by a solid-state reaction in a vacuum-sealed tube shows an electron mobility of 2320 cm^2^/V/s,^[^^24^^]^ which is much larger than the 200 cm^2^/V/s measured for solution-processed material.^[^^69^^]^

The electron mobility of polycrystalline CH_3_NH_3_PbI_3_ films compares favorably to that of films of other materials used as absorbers in solar cells. It is larger than the thin-film mobility of polymers (10^−7^–1 cm^2^/V/s)^[^^70^^,^^71^^]^ and colloidal quantum dots (10^−3^–1 cm^2^/V/s),^[^^72^^]^ and it is comparable with that of CdTe (10 cm^2^/V/s),^[^^73^^]^ CIGS and Cu_2_ZnSnS_4_ (CZTS) (10–10^2^ cm^2^/V/s),^[^^74^^,^^75^^]^ and polycrystalline Si (40 cm^2^/V/s).^[^^76^^]^ Perhaps in its monocrystalline form, the intrinsic mobility might be similar to that of monocrystalline Si (10^2^–10^3^ cm^2^/V/s)^[^^77^^]^ or GaAs (>10^3^ cm^2^/V/s),^[^^78^^]^ but further studies must be performed on single crystals synthesized using a variety of methods. Even in polycrystalline form, hybrid perovskites’ inexpensive processing and tolerance to defects offer a significant advantage over conventional semiconductors.

As is the case for other polycrystalline semiconductors, electrical properties in hybrid perovskites are likely correlated with the film's morphology. For instance, the dark and light conductivities of CH_3_NH_3_PbI_3−*x*_Cl_*x*_ deposited on a planar scaffold or on mesostructured aluminum oxide are quite different.^[^^79^^]^ This difference has been attributed to an increase in the perovskite's Fermi level in the mesostructured scaffold either through more surface iodide vacancies or through electrostatic gating from the aluminum oxide. Also, the role of grain boundaries in conduction through perovskite films has not been thoroughly explored, although passivation of grain boundaries with PbI_2_ has been correlated to increased radiative lifetimes.^[^^80^^]^ Inspired by the literature on organic solar cells, solvent annealing has been applied to CH_3_NH_3_PbI_3_ solar cells to increase the grain size of the films to ~1 μm.^[^^35^^]^ Such processing results in an increase in the photovoltaic performance and radiative lifetime.

### Extrinsic electrical properties of operating solar cells

To investigate the electrical properties of perovskites in devices, techniques such as impedance spectroscopy (IS)^[^^81^^,^^82^^]^ and electron beam-induced current (EBIC)^[^^83^^,^^84^^]^ have been applied. IS is typically used to identify the frequency dependence of capacitance, to measure charge diffusion lengths and lifetimes, and to investigate carrier trapping and recombination. An equivalent circuit that has been used successfully to analyze DSSCs can be adapted to identify characteristics of perovskite solar cells; however, the model should be taken from a solid-state DSSC, which also uses a hole-transport layer, such as 2,2′,7,7′-tetrakis(N,N-di-p-methoxyphenylamine)-9,9′-spirobifluorene (spiro-OMeTAD), instead of redox ions in a liquid electrolyte.^[^^81^^]^ Capacitance values extracted from IS of perovskite solar cells show evidence of significant charge accumulation in the perovskite in the dark and under illumination,^[^^85^^]^ which could arise from trap states within the material. Such an interpretation is consistent with Kelvin probe force microscopy measurements that found long-lived trapped charges within the perovskite after illumination that accumulate because of poor transport through the hole-transport layer.^[^^86^^]^ These trapped charges are one possible source of the hysteresis often seen in the operation of perovskite solar cells (discussed below in Section 5.3).

In addition to the capacitance, the carrier diffusion length can also be derived from IS and has been estimated to be about ~1 μm for CH_3_NH_3_PbI_3−*x*_Cl_*x*_.^[^^82^^]^ This result is similar to values obtained using EBIC (1.5–1.9 μm)^[^^83^^]^ and PL quenching (~1 μm).^[^^56^^]^ The carrier diffusion length in CH_3_NH_3_PbI_3−*x*_Cl_*x*_ is comparable with or longer than that of other polycrystalline semiconductors with direct band gaps used in solar cells, such as CZTS (350–450 nm),^[^^74^^]^ CIGS (300–900 nm),^[^^75^^]^ and CdTe thin films (0.4–1.6 μm).^[^^87^^]^ It is also much longer than the typical excitonic diffusion lengths in other solution-processed technologies such as conducting polymers (5–10 nm),^[^^88^^]^ and films of colloidal quantum dots (100 nm).^[^^89^^]^

### Origin of electrical hysteresis in hybrid perovskites

**Figure 4.**
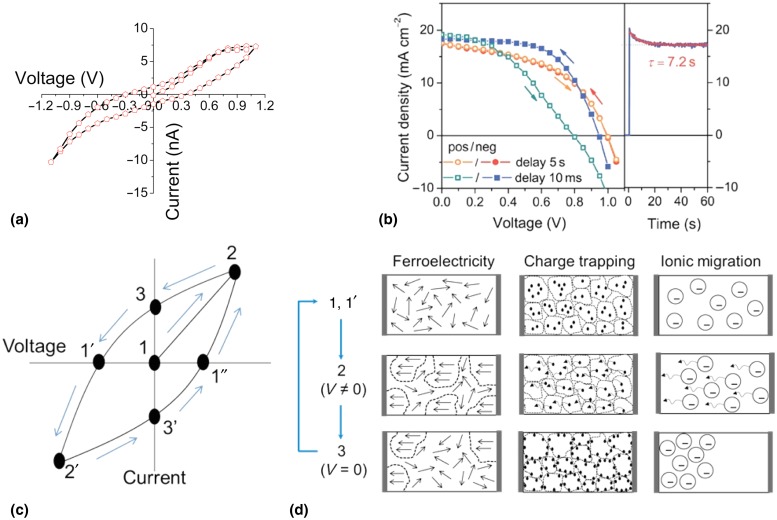
Hysteresis in the electrical transport in hybrid perovskites. (a) *I–V* measurement taken at room temperature on a single crystal of CH_3_NH_3_PbI_3_ exhibiting pronounced hysteresis. (b) The *I–V* characteristic (left) of a perovskite solar cell depends upon the rate at which the voltage is scanned: in this specific case, more hysteresis is observed for the faster scan rate. The photocurrent stabilizes with a long time constant of 7.2 s (right). Schematic hysteretic *I–V* curve (c) and phenomena proposed for its origin (d). The as-synthesized perovskite exists in a disordered state (1). When a bias is applied (2), the electric field creates a change in the material, as depicted in (d) for the three proposed mechanisms. Upon return of the field back to 0 V (3), the arrangement of dipoles, trapped charges, or ions has been altered from the initial state, so current continues to flow as this configuration relaxes, creating hysteresis. The material returns to its initial state (1′) upon application of a negative bias. The same process with reversed polarity occurs for points 2′, 3′, and 1″. Panel (a) is reproduced with permission from the supporting information from Ref. 24. Copyright 2013, American Chemical Society. Panel (b) is reproduced with permission from Ref. 92. Copyright 2014, Royal Society of Chemistry.

In the dark and light, CH_3_NH_3_PbI_3_ devices exhibit distinct hysteresis behavior in both their resistivity versus temperature dependence (*R–T* characteristic) and current versus voltage dependence [*I*–*V* characteristic, Fig. 4(a)].^[^^24^^]^ While the hysteresis on the *R*–*T* curve has been associated with a structural phase transition in the perovskite, the cause of the hysteresis in the *I*–*V* curve at room temperature is still unclear (Fig. 4). Additionally, CH_3_NH_3_PbI_3_ exhibits a very slow transient (~10–100 s) to reach a steady state in current or voltage [Fig. 4(b)].^[^^90^^–^^92^^]^ This time scale has been verified from the characteristic times measured by transient photovoltage,^[^^93^^]^ transient PL,^[^^93^^]^ and impedance spectroscopies.^[^^81^^,^^85^^,^^93^^]^ Some variation in the time scale results from changing the crystal growth process. For example, the single-step solution deposition gives transient times that are three orders of magnitude slower than the two-step process.^[^^93^^]^ Several explanations have been proposed for this behavior, including ferroelectricity, accumulation of charges at grain boundaries, filling of interface or surface trap states, and ionic migration [Figs. 4(c) and 4(d)].^[^^91^^]^ Distinguishing between these mechanisms is an area of ongoing research.

Understanding ferroelectricity within a hybrid perovskite requires consideration of the crystal symmetry as well as the incorporated CH_3_NH_3_^+^ cations. From a structural perspective, the tetragonal phase of CH_3_NH_3_PbI_3_ does allow for a polar ferroelectric distortion, whereas the cubic phases of the chloride and bromide perovskites do not. Unlike conventional oxide perovskites, however, hybrid perovskites include a molecular dipole, CH_3_NH_3_^+^, which reorients freely on the picosecond timescale at room temperature.^[^^18^^–^^20^^]^ The molecule is not spherically symmetric like an atom, but its unimpeded reorientations at room temperature impart a pseudo-spherical symmetry. It is hypothesized that application of an external electric field stabilizes a state in which the dipoles are aligned and the inorganic lattice is distorted; therefore, the long-time constant in the electrical measurements arises from the slow polarization of the inorganic lattice that must occur because of its interaction with the organic cations.^[^^90^^]^ It is possible that such an electric-field-induced structural change might be measured by in situ XRD, as has been demonstrated in certain oxide perovskites.^[^^94^^]^ If this mechanism is correct, the hysteresis should depend on the magnitude of the dipole moment of the organic cation and its coupling to the halide cage via hydrogen bonding; perovskites synthesized from various cations with different dipoles could be tested and compared.^[^^95^^]^

Some experimental evidence supports the existence of ferroelectricity in CH_3_NH_3_PbI_3_. Ferroelectric domains have been mapped using piezoforce microscopy on solution-processed CH_3_NH_3_PbI_3_.^[^^44^^]^ Also, ferroelectricity would explain the large static dielectric constant (*ε*_0_) of CH_3_NH_3_PbI_3_ of ~1000, although grain boundaries could also be responsible for it.^[^^96^^]^ In typical ferroelectrics, a discontinuity in the dielectric constant marks the ferroelectric to paraelectric phase transition. For hybrid perovskites such a singularity has been observed only at the phase transition at which the CH_3_NH_3_^+^ cations become ordered,^[^^19^^]^ which is well below room temperature. These measurements were performed at 20 Hz, however, so it is likely that they were too fast to allow the polarization change, which occurs on the 10–100 s timescale, to be realized, essentially removing this contribution to the dielectric constant. Since the change in dielectric constant at the phase transition is one of the hallmarks of a ferroelectric material, repetition of these measurements at sufficiently low frequencies would do much to clarify this issue.

If CH_3_NH_3_PbI_3_ is ferroelectric, knowledge gained from the extensive study of oxide ferroelectrics can inform the interpretation of effects seen in perovskites. For example, it has been proposed that ferroelectric domains could be responsible for the excellent performance of perovskite photovoltaics by aiding in charge separation.^[^^95^^]^ A similar mechanism has been demonstrated experimentally in BiFeO_3_^[^^97^^]^ and was later explained in detail.^[^^98^^]^

In addition to ferroelectricity, charge accumulation at grain boundaries, ionic migration, and trapping of charges at surfaces or interfaces, have also been proposed as possible sources of the hysteresis.^[^^91^^]^ The reported large static dielectric constant is consistent with effects from grain boundaries,^[^^96^^,^^99^^]^ and measuring the dielectric response of single crystals with varying contact geometries to avoid artifacts from trapped charges at electrode interfaces^[^^99^^,^^100^^]^ would provide insight into the role of grain boundaries. Also, no hysteresis was detected in solvent-annealed solar cells with grain sizes large enough to permit pseudo-single crystal transport in the through-plane direction.^[^^35^^]^ Such an observation cannot rule out a ferroelectric mechanism, however, because the switching of ferroelectric domains is known, at least in oxide perovskites, to be highly sensitive to grain size and morphology.^[^^101^^]^ In the case of ionic migration, a first step would be to determine the contribution of ionic conduction by comparing frequency-dependent AC and DC resistivity measurements, which would suggest whether either halide anions or CH_3_NH_3_^+^ cations are mobile and available to screen the electric field. Related inorganic perovskites, CsPbCl_3_ and CsPbBr_3_ have been determined to be halide-ion conductors, as are the precursor halides PbCl_2_ and PbBr_2_,^[^^102^^]^ which have sometimes been found by XRD to contaminate the perovskite films. Cycling of a large bias (±2.5 V) applied to perovskite solar cells has been shown to produce switchable photocurrent and photovoltage, possibly explained by the reversible migration of vacancies that leads to a bias-induced p–i–n doping structure.^[^^103^^]^ Hysteresis in perovskites has also been shown to be sensitive to the contacting scheme employed for the perovskite film, which suggests that it might be related at least in part to interfacial trap states or to the filling and emptying of bulk trap states.^[^^91^^]^

## Optical properties

Understanding the optical response of a material is crucial for optoelectronic applications such as photovoltaics, but it can also provide insight into the electronic and chemical structure.

### Optical constants

**Figure 5.**
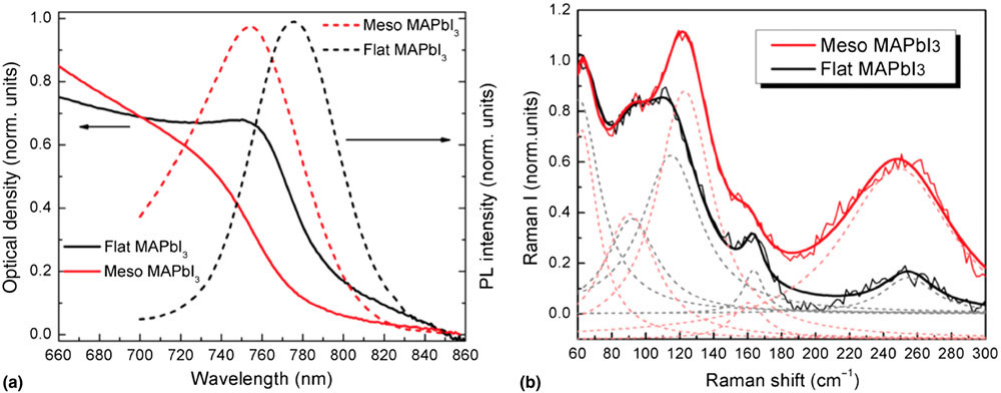
Absorption and PL spectra (a) and resonant Raman spectra (b) of CH_3_NH_3_PbI_3_ deposited on flat substrates and in mesoporous substrates. Clearly the optical properties of the material depend sensitively on the material's structure, which is determined in part by the substrate on which it is deposited. Solid lines in (b) are the sums of the individual peaks (dotted lines) used to fit the spectra. The figure is reproduced with permission from Ref. 52. Copyright 2014, American Chemical Society.

Although tuning the band gap of perovskites is well documented, a more detailed understanding of these materials’ optical properties awaits further research. For instance, dielectric constants in the ultraviolet, visible, and near-IR spectral regions are critical to understanding the optical response of perovskites and also to calculating their absorption and emission properties when incorporated into optoelectronic devices. Progress in this area has been hindered by the difficulty of producing continuous films of sufficient smoothness^[^^14^^]^ to avoid measurement artifacts from spectroscopic measurements of reflectance, transmittance, and ellipsometry. Recent work using ellipsometry to characterize films optimized to reduce roughness, and which accounts for remaining roughness via modeling, has produced the most reliable optical constants of CH_3_NH_3_PbI_3_,^[^^104^^]^ although they still remain to be independently confirmed. These latest values differ considerably from previous preliminary measurements on CH_3_NH_3_PbI_3_ films,^[^^39^^,^^105^^]^ which is not surprising since earlier measurements did not account for the film's morphology. Quantitative absorption coefficients have been determined from the absorption of CH_3_NH_3_PbI_3_ films on quartz^[^^39^^]^ and glass,^[^^25^^]^ yielding values of ~10^4^ cm^−1^ near the band edge, but no detailed data of the film's morphology has been provided and no corrections for the surface's inhomogeneity have been applied; consequently, the reported values are only preliminary, but they are consistent with the absorption coefficients calculated based on the optical constants of CH_3_NH_3_PbI_3_.^[^^104^^]^ Additionally, the absorption spectrum for CH_3_NH_3_PbI_3_ when it is deposited within a mesoscopic template differs from that of perovskite deposited on planar substrates, which has been attributed to changes in the crystallite morphology that affect the optical transitions [Fig. 5(a)].^[^^52^^]^ Band structure calculations indicate that this change in morphology affects the dipole screening of the excitonic transition.^[^^64^^]^ Such differences further complicate accurate determination of the absorption coefficient and other optical parameters of perovskite films, so control over the materials properties of perovskites is of the greatest importance for characterization and applications.

### Excitons

The role of excitons in perovskites has been a topic of considerable debate. Recent studies indicate, however, that no significant population of excitons is present in photovoltaics made from CH_3_NH_3_PbI_3_, whose exciton-binding energy has generally been reported between 20 and 50 meV and is therefore comparable with the thermal energy at room temperature (*k*_B_*T* = 26 meV).^[^^53^^,^^106^^]^ These values have been obtained by fitting temperature-dependent absorption spectra using the measured^[^^107^^]^ reduced mass of the exciton. Estimating the excitonic radius from the binding energy, however, requires knowledge of the appropriate^[^^65^^]^ dielectric constant, which continues to be the subject of debate.^[^^105^^]^ Although the population of excitons is small in photovoltaics and remains small even at the higher excitation densities required for stimulated emission,^[^^106^^]^ the excitonic transition significantly enhances the absorption of hybrid perovskites near the band edge; consequently, the electronic band gap taking this into account is 1.65 eV for CH_3_NH_3_PbI_3_.^[^^106^^]^ The density of excitons and the associated effects should be greater in perovskites with higher band gaps, such as CH_3_NH_3_PbBr_3_, whose binding energy has been estimated to be 76 meV.^[^^107^^]^

### Photoluminescence

PL has been observed in perovskites of pure and mixed compositions. PL efficiency depends strongly upon pump fluence. At low excitation intensities, trapping of photogenerated charges competes effectively with direct radiative recombination of electrons and holes, reducing luminescence.^[^^8^^,^^106^^,^^108^^,^^109^^]^ As the excitation increases, these traps are filled and radiative electron–hole recombination dominates. In this regime, studies have confirmed low trap-induced recombination and two-body recombination dynamics over a range of pump intensities.^[^^110^^,^^111^^]^ High PL efficiency, ~70% for CH_3_NH_3_PbI_3−*x*_Cl_*x*_,^[^^8^^,^^106^^]^ and ~17–30% for CH_3_NH_3_PbI_3_,^[^^39^^,^^106^^]^ is achieved because amplified spontaneous emission begins at a threshold of only ~10 μJ/cm^2^, which corresponds to an injected carrier density of ~10^18^ cm^−3^ or three orders of magnitude greater than the excitation expected under solar illumination.^[^^39^^,^^106^^]^ At higher pumping, the PL efficiency falls as Auger recombination becomes more competitive at the higher carrier densities.^[^^14^^,^^39^^,^^106^^]^

PL lifetime measurements have been reported for CH_3_NH_3_PbI_3_ and CH_3_NH_3_PbI_3−*x*_Cl_*x*_, with longer lifetimes exhibited by the latter. In general, it is difficult to compare lifetimes directly unless they are measured at the same pump fluence, and the wide range of reported lifetimes for each material is likely due to a combination of varying excitation density and synthetic procedures. The PL lifetime of CH_3_NH_3_PbI_3_ has been reported between 3 and 18 ns,^[^^39^^,^^50^^,^^112^^]^ while CH_3_NH_3_PbI_3−*x*_Cl_*x*_ exhibits considerably longer lifetimes from 91 to 341 ns at low pump fluences.^[^^43^^,^^56^^,^^108^^,^^110^^]^ Reports that measure both materials under the same excitation conditions indicate an increase from 18 to 91 ns^[^^43^^]^ and 10 to 273 ns.^[^^56^^]^ This increase in lifetime correlates well with the factor of ten increase in carrier diffusion length, indicating that lifetime rather than mobility is improved by the addition of Cl^−^; measurements have confirmed that the mobility increases only slightly from 8 to 11.6 cm^2^/V/s for CH_3_NH_3_PbI_3_ and CH_3_NH_3_PbI_3−*x*_Cl_*x*_, respectively.^[^^55^^]^ For solution-processed polycrystalline semiconductors, such lifetimes are surprising, especially considering that essentially nothing has been done to reduce recombination at surfaces or grain boundaries. Passivation of grain boundaries with PbI_2_^[^^80^^]^ or Lewis bases^[^^108^^]^ or using solvent annealing^[^^35^^]^ to increase grain size have improved the PL lifetime, with lifetimes reaching 2 μs in the case of passivation with Lewis bases. Such long radiative lifetimes in a semiconductor with a direct band gap have been seen in undoped and surface-passivated GaAs films.^[^^113^^]^ In this case, photon recycling causes the PL lifetime to be limited by surface recombination rather than radiative recombination. Considering that the perovskites exhibit clear self-absorption in their PL^[^^106^^]^ and lasing spectra,^[^^39^^]^ it would not be surprising for photon recycling to play a role in their excited state dynamics when pathways of non-radiative decay are adequately suppressed.

### IR and Raman spectroscopy

Probing the band gap with visible light offers information about the electronic structure of these compounds, but IR spectroscopy can provide a more detailed chemical understanding. At room temperature, CH_3_NH_3_PbI_3_ is tetragonal, while CH_3_NH_3_PbBr_3_ and CH_3_NH_3_PbCl_3_ are cubic. Symmetry of the lattice in principle precludes Raman-active modes for the cubic crystals,^[^^114^^,^^115^^]^ although a weak, broad band has been observed about 66 cm^−1^ in CH_3_NH_3_PbCl_3_ that is perhaps allowed by disorder within the material.^[^^114^^]^ For tetragonal CH_3_NH_3_PbI_3_, a recent study comparing the resonant Raman spectrum with density-functional theory (DFT) calculations based on a harmonic approximation has identified modes below approximately 100 cm^−1^ related to the inorganic octahedron as well as higher energy modes indicative of the disorder of the CH_3_NH_3_^+^ cations [Fig. 5(b)].^[^^52^^,^^116^^]^ Although calculations and comparison with the literature of related compounds have informed the interpretation of the spectrum, further investigation of how these modes shift with structural changes in the crystal can be used to confirm the assignments. The temperature-dependent IR spectra of CH_3_NH_3_^+^ within all three perovskites were investigated^[^^117^^]^ and supported the earlier conclusion that the cations remain disordered until phase transitions below room temperature.^[^^21^^]^ A detailed description of the IR spectrum below 850 cm^−1^, where the inorganic bands would be expected to appear and have been documented in CsPbCl_3_,^[^^118^^]^ has not been reported for the hybrid perovskites. Once fundamental assignments of modes have been confirmed, Raman and IR spectroscopy can become tools with which to study structure–function relationships in perovskites. In particular, spatial mapping of Raman modes has the potential to offer a richer understanding of the inhomogeneity of perovskite films with sub-micron spatial resolution.

## Emerging applications

**Figure 6.**
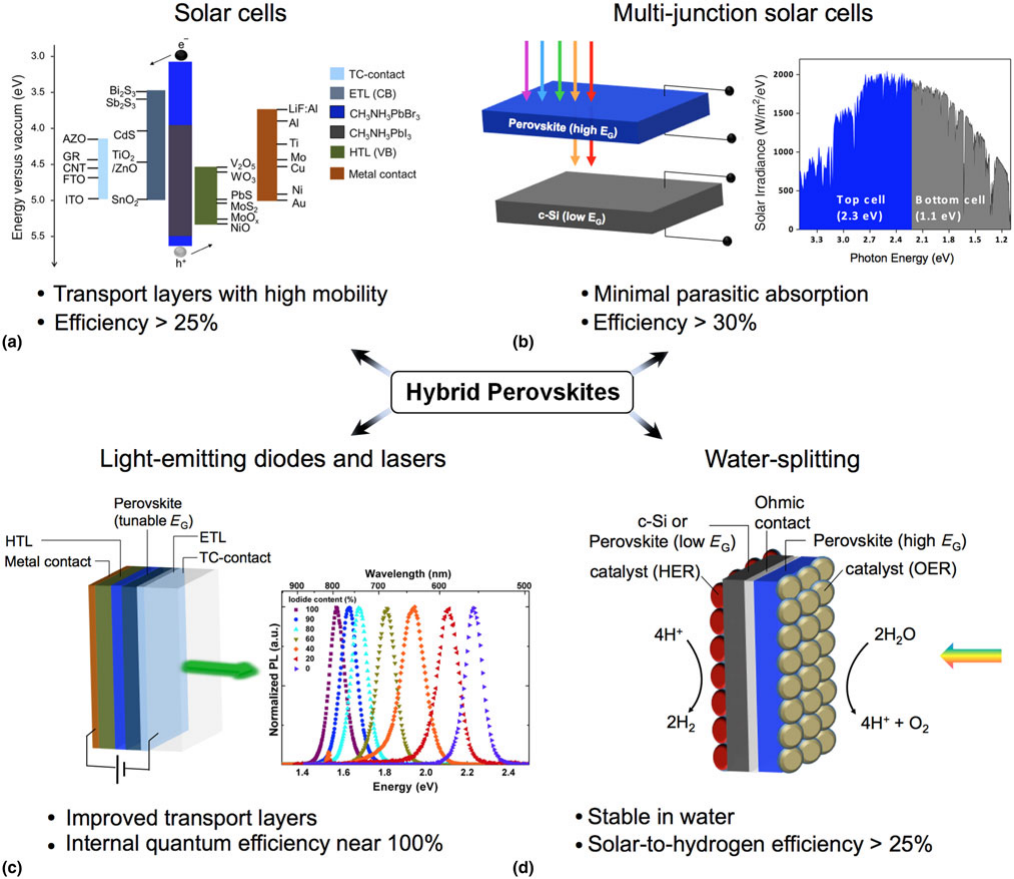
Targets for emerging applications of hybrid perovskites. The success of single-junction photovoltaics has paved the way for other applications of hybrid perovskites. Improvements can be made in the contacting of perovskites for higher efficiency solar cells (a), and perovskites can be combined with crystalline silicon for an efficient yet inexpensive tandem solar cell (b). Additionally, emission of light in the form of LEDs or lasers holds much promise (c). If the perovskites’ instability in water can be overcome, perhaps through encapsulation, then their tunable band gap makes them an attractive option for solar water-splitting (d). Transparent-conducting contact, TC-contact; electron-transport layer, ETL; hole-transport layer, HTL; valence band, VB; conduction band, CB; aluminum-doped ZnO, AZO; graphene, GR; carbon nanotubes, CNT; fluorine-doped and indium tin oxide, FTO and ITO; hydrogen and oxygen evolution reaction, HER and OER. The PL spectra included in the figure are reproduced with permission from Ref. 40. Copyright 2014, American Chemical Society.

Although the predominant application of hybrid perovskites has so far been in single-junction photovoltaics, a variety of other technologies can benefit from the unique properties of these materials (Fig. 6).

### Solar cells: better materials for transport layers and contacts

Current high-efficiency perovskite solar cells can be further optimized using appropriate contacts to extract photogenerated charges efficiently. Present designs typically sandwich the perovskite between a hole-transport layer and electron-transport layer, which are then contacted by a metal or a transparent conducting contact. Important characteristics for the selective layers include: (1) suitable band alignment that permits the flow of one carrier and blocks the other; (2) electrical conductivity and in particular a charge mobility that is comparable with that of the perovskite; (3) a homogeneous and intimate contact with the perovskite, which might require a conformal deposition technique; (4) chemical stability against reactions with the perovskite; and (5) transparency of at least one contact to allow the absorption of light.

To create carrier-selective contacts, the use of metal oxides and metal sulfides is promising because they are stable in air and have charge mobilities higher than those of the organic semiconductors currently being used. In addition, metal oxides and sulfides have been used in most commercial thin-film solar cells and have proven better in most cases for organic photovoltaics (OPVs). Typical electron-transport layers in perovskite solar cells are metal oxides such as TiO_2_, ZnO, Al_2_O_3_, and ZrO_2_ or fullerene derivatives such as 6,6-phenyl C_61_ butyric acid methyl ester (PCBM). Since metal sulfides have electron mobilities that are two to three orders of magnitude higher than those of the standard oxides, they are an attractive alternative. Candidates include CdS, ZnS, Bi_2_S_3_, MoS_2_, and Sb_2_S_3_.^[^^119^^]^ Care must be taken to keep the layers thin because these metal sulfides absorb visible light, with the exception of ZnS.

The mobility of the hole-transport layer also needs to be improved, and metal oxides and sulfides can contribute here as well. Highly efficient perovskite cells currently use organic semiconductors with high work functions, such as spiro-OMeTAD,^[^^51^^]^ poly(3-hexylthiophene) (P3HT),^[^^120^^]^ poly(3,4-ethylenedioxythiophene)-polystyrenesulfonic acid (PEDOT:PSS),^[^^121^^]^ or other polymer derivatives such as carbazoles,^[^^122^^]^ polyfluorenes,^[^^123^^]^ pyrenes,^[^^124^^]^ and triarylamines.^[^^125^^]^ All of them have much lower hole mobility (μ_h_ = 10 ^−^^6^–10^−2^ cm^2^/V/s) than CH_3_NH_3_PbI_3_, except for PEDOT:PSS (μ_h_ = 1 cm^2^/V/s); however, PEDOT:PSS is not ideal because of its inability to block electrons, acidity, and tendency to absorb water. The latter two disadvantages are often associated with degradation in OPVs^[^^126^^,^^127^^]^ and would likely be similarly detrimental to perovskite solar cells. In contrast, MoS_2_ can have carrier mobilities in excess of 100 cm^2^/V/s and is chemically less reactive because of its layered crystal structure.^[^^128^^]^ MoS_*x*_Se_1−*x*_ has been successful as a hole contact in CIGS solar cells, and its valence band is also expected to align favorably with that of the perovskites. Moreover metal oxides such as NiO^[^^129^^]^ and MoO_*x*_^[^^130^^,^^131^^]^ have also been used with perovskites, and other metal oxides that have higher valence bands, such as V_2_O_5_^[^^131^^]^ and WO_3_^[^^132^^]^ are of interest because of their successful application in organic solar cells. The hole-transport layer for CH_3_NH_3_PbI_3_ could also be formed from other halide perovskites such as CsSnI_3_ or CH_3_NH_3_SnI_3_ since tin halide perovskites have very high hole mobilities (μ_h_ = 10^2^–10^3^ cm^2^/V/s).^[^^133^^]^

Ideally, the transport layers should be able to make homogeneous contact with the perovskite layer to ensure efficient collection of charges. Optimizing the surface chemistry of the layers and the perovskite could induce a preferential orientation for growth of the film and therefore yield better contact between the perovskite layer and its scaffold.

The transport layer should also be chemically inert. For example, mesoporous TiO_2_ exhibits pronounced degradation under ultraviolet light, which creates oxygen vacancies on the TiO_2_ surface that are deep traps for photogenerated electrons.^[^^134^^]^ Coating TiO_2_ with ZrO_2_^[^^135^^]^ or Sb_2_S_3_^[^^119^^]^ or replacing it with a non-conductive scaffold such as Al_2_O_3_^[^^136^^]^ greatly improves the stability of the device. Clearly, engineering the interface between the perovskite and its contact layers is important in improving performance and overcoming device instability.

Lastly, developing new transparent contacts is also important. Fluorine- and indium-doped tin oxide (FTO and ITO), the two most popular transparent conducting oxides currently in use, will likely be replaced with less expensive materials. Possibilities include aluminum-doped ZnO, graphene, thin metal contacts, and networks of carbon nanotubes or metal nanowires.

Testing a variety of interfacial layers and contact materials does pose challenges because perovskites cannot withstand the conditions of many conventional processes used to produce thin films. Perovskites’ decomposition at relatively low temperatures (~200 °C)^[^^48^^]^ precludes many vapor-phase chemical deposition processes, and their dissolution in water and some polar organic solvents limits solution-based processes such as chemical bath deposition, sol–gel chemistry, and spincoating. Additionally, the organic component of the perovskite is likely to be damaged by plasma-assisted processes or oxidizing environments. Currently, the most commonly employed methods for depositing films on perovskites are thermal and electron-beam evaporation and spincoating from non-polar solvents. While deposition of the transport layer beneath the perovskite can be achieved using any method compatible with the underlying electrode, methods for depositing layers on top of the perovskite are restricted.

### Multi-junction photovoltaics

The overall cost of electricity generated from a photovoltaic system is determined by the expenses associated with the solar module, the balance-of-system components, and the system's installation and maintenance. Since the cost of the module is small relative to other costs, improving the module's efficiency while only marginally increasing its cost is the best way to reduce the cost of generated energy. The efficiency of a single-junction solar cell is limited by the Shockley–Queisser limit, which includes losses from transmitted below-band-gap photons and from thermalization of hot photogenerated carriers.^[^^137^^]^ The concept of multi-junction photovoltaics is to improve the efficiency by minimizing these losses. Within a stack of solar cells with decreasing band gaps, photons with higher energies are converted by the high-band-gap solar cells on top, while the photons with lower energy pass through to be converted by the lower-band-gap solar cells. Conventional fabrication of multi-junction photovoltaics is costly because the stacked crystalline absorber layers must be lattice matched and are produced by expensive epitaxial growth processes. Incorporating defect-tolerant materials that give devices with high efficiencies, such as perovskites, into the multi-junction concept has the potential to be much less expensive than current III–V technology and might improve the overall efficiency. This goal can be achieved by stacking perovskite cells on well-established photovoltaic cells, such as those made from crystalline Si (c-Si), GaAs, CIGS, or CdTe. The perovskite cell can be used as the top cell because its band gap can be tuned to transmit sufficient light to the cell beneath.

There are two basic designs of the multi-junction concept: two-terminal (two stacked cells that are connected electrically in series) and four-terminal (two stacked cells that are electrically independent). The four-terminal design has advantages over the two-terminal design including no requirement of current matching, no need for a tunnel junction or recombination layer, freedom to choose any combination of materials without the need for lattice matching, and simpler prototyping. The primary disadvantage is the parasitic absorption, which is any absorption that cannot lead to photocurrent, that occurs in the four-terminal cell's extra contacts.

The perovskite cells can be mechanically stacked in a four-terminal tandem cell with current established c-Si solar cells to yield higher efficiencies than either of the single cells. For example, adding a semi-transparent perovskite solar cell to low-performing silicon and CIGS solar cells has already been shown to increase the cell's total efficiency.^[^^138^^]^ Looking forward, 30%-efficient tandem cells can be achieved for CH_3_NH_3_PbI_3_ (*E*_g_optical_ = 1.6 eV)/c-Si and CH_3_NH_3_PbBr_3_ (*E*_g_optical_ = 2.2 eV)/c-Si pairings under one-sun illumination using a minimum 21.0%-efficient CH_3_NH_3_PbI_3_ cell or a 13.2%-efficient CH_3_NH_3_PbBr_3_ cell on top and a 25%-efficient c-Si cell below.^[^^139^^]^ The maximum attainable efficiencies for optimized versions of these tandem devices are estimated at 31.6% and 32.5%, respectively. These estimates of combined efficiency are based on the absorption coefficient, diffusion length, and radiative efficiency of CH_3_NH_3_PbI_3_, which are taken to be 10^4^ cm^−1^, 100 nm, and 10^−2^%, respectively. An efficiency of over 30% for hybrid perovskite/c-Si tandem devices is realistic, since the maximum efficiency to date for solar cells based on CH_3_NH_3_PbI_3_ is 20.1%^[^^1^^]^ and 10.4% for solar cells made from CH_3_NH_3_PbBr_3_.^[^^34^^]^ Also, low-temperature processing of the hybrid perovskite cells allows them to be produced on top of other cells without damaging them. Combining solution-processed solar cells such as perovskites with conventional solar cells can increase their efficiency without significantly increasing the cost of production.

The main challenge in fabricating a successful tandem device is minimizing parasitic absorption. The top cell's bottom contact must be semi-transparent so that light reaches the cell underneath. Also, the material used for the top cell itself should have minimal sub-band-gap absorption, for instance from trap states or compositional inhomogeneity. Recent measurements of the perovskites’ sub-band-gap absorption using photothermal deflection spectroscopy indicate that CH_3_NH_3_PbI_3_ performs well in this regard, exhibiting a sharp decrease in absorption below the optical band gap.^[^^40^^]^ Mixtures of iodide and bromide, however, show comparatively greater sub-band-gap absorption that can perhaps be reduced by improved synthetic techniques.

### Building-integrated photovoltaics

Integrating semitransparent solar cells into windows is one highly attractive application for perovskite solar cells. Perovskites are one of the few materials that can deliver tunable semitransparency while maintaining high-power-conversion efficiency. Their success is enabled by their unique morphology: micron-sized islands of perovskite with gaps between them. The micron-sized islands are thick enough to absorb most of the visible light, but the gaps are sufficiently large to allow transmission. Controlling the morphology and thickness of the islands along with the sizes of the gaps produces a device with acceptable efficiency (reaching 6.4% so far) and up to 60% transmittance from 400 to 750 nm.^[^^140^^]^ Similar transparency has also been demonstrated using DSSCs^[^^141^^]^ and OPVs^[^^142^^]^; however, IR-absorbing dyes and polymers exhibit non-uniform absorption of visible light that tends to make these devices appear tinted rather than color-neutral.

A possible target for implementation is to improve the efficiency to 12% while maintaining the existing 60% transmittance. A step toward this goal might come from replacing the gold electrode on hybrid perovskite solar cells with conductive materials that are more transparent, such as graphene or meshes of carbon nanotubes or silver nanowires. Although this approach is technically challenging, it has been shown to be promising in OPVs^[^^143^^,^^144^^]^ and perovskite tandem solar cells.^[^^138^^]^ Additionally, maximizing the photon absorption in the invisible regions of the solar spectrum by engineering the band gap or the optical density of states of the perovskites is also an interesting prospect.

### Light emission and detection

The observation of PL in pure and mixed-composition perovskites makes these materials of great interest for optoelectronic applications beyond photovoltaics. Light-emitting diodes,^[^^6^^,^^7^^]^ lasers,^[^^8^^–^^10^^]^ and photodetectors^[^^11^^,^^12^^]^ have already been demonstrated, yet the continued success of these applications hinges upon the development of appropriate materials. As in the case of photovoltaics, selection of contacts and transport layers with suitable conductivity and band alignment for the injection of charges is a challenge, and minimizing series resistance from these layers is crucial since light-emitting diodes can operate at current densities that are orders of magnitude larger than the current densities in solar cells. Because of the full tunability of the emission of perovskites, tandem stacked light-emitting diodes, such as those currently produced using organic light-emitting diodes,^[^^145^^]^ are an interesting possibility for a white-light source. As an alternative, perhaps a microscopically phase-segregated mixture of the perovskites tuned across the visible spectrum could yield white-light emission, given the right contacting design.

### Solar water-splitting

Perovskites also hold promise for the conversion of sunlight directly into chemical fuels, a process called artificial photosynthesis. Perovskite solar cells have already been used to split water into O_2_ and H_2_,^[^^5^^]^ although they were not immersed in the electrolyte. Two perovskite solar cells connected in series were required to achieve the photovoltage necessary to overcome the thermodynamically required minimum voltage of 1.23 V for splitting water and the additional 0.1 and 0.3 V for kinetically driving the H_2_ and O_2_ evolution reactions.^[^^146^^]^ The maximum solar-to-hydrogen conversion efficiency demonstrated by this system was 12.3%, which is close to the most notable example of water-splitting using GaInP/GaAs tandem cells that achieved 12.4%.^[^^147^^]^ Achieving a solar-to-hydrogen conversion efficiency of over 20% is very realistic by using a tandem architecture, for example by combining a perovskite top cell with a c-Si or CIGS bottom cell. The appropriate selection of catalysts is also critically important in order to reduce the overpotential of the chemical reactions. In terms of efficiency and cost-effectiveness, the use of perovskites has already been exceptional. The greatest remaining challenge is how to overcome the instability of perovskites since they show degradation in performance after only hours of operation and are typically soluble in water. Encapsulation of the perovskite in a conductive oxide such as TiO_2_ is one strategy toward achieving stability in water since this approach has already been shown to stabilize other semiconductor photoelectrodes.^[^^148^^]^

## Challenges

Over the course of the development of perovskites, several key challenges have arisen that must be addressed if these materials are to make a technological impact.

### Stability and mechanisms of degradation

**Figure 7.**
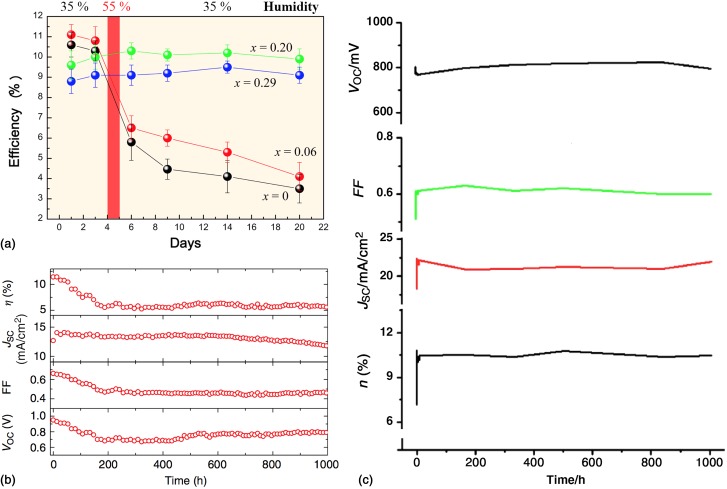
(a) Comparison of the stability of solar cells made from mixed perovskites CH_3_NH_3_Pb(I_1−*x*_Br_*x*_)_3_. Devices were stored in air without encapsulation and tested periodically. On the fourth day the devices were exposed to 55% humidity to evaluate the effect of water on their stability. (b) Stability of a solar cell made from CH_3_NH_3_PbI_3−x_Cl_x_ on an alumina scaffold under continuous illumination of 76.5 mW/cm^2^ (~3/4 one-sun intensity) at 40 °C. The device was encapsulated in a nitrogen environment. (c) Stability over 42 days (1008 h) of a perovskite solar cell made from mixed organic cations CH_3_NH_3_PbI_3_ and 5-ammoniumvaleric acid. The unsealed device was continuously illuminated under one-sun, A.M. 1.5 conditions in air, although the carbon back contact is expected to provide some encapsulation. Panel (a) is reproduced with permission from Ref. 38, copyright 2013, American Chemical Society. Panel (b) is reproduced with permission from Ref. 134. Copyright 2013, Nature Publishing Group. Panel (c) is adapted with permission from the supplementary materials from Ref. 135. Copyright 2013, American Association for the Advancement of Science.

By far, the chief concern raised in the development of perovskites into practical technology is their long-term stability. At present, moisture and heat have been identified as triggers for instability in CH_3_NH_3_PbI_3_. Since hybrid perovskites are ionic solids, they are unstable in the presence of many polar solvents, most notably water. Films of CH_3_NH_3_PbI_3_ degrade when exposed to high humidity >50%,^[^^38^^]^ while such degradation was not observed for CH_3_NH_3_Br_3_; consequently, the device performance of CH_3_NH_3_PbI_3_ solar cells decreased while that of CH_3_NH_3_Pb(I_1−*x*_Br_*x*_)_3_ with *x* = 0.20 and 0.29 remained relatively stable over a period of 20 days of storage [Fig. 7(a)]. Although the exact degradation mechanism is not known, it is postulated that water accepts the proton from the CH_3_NH_3_^+^, yielding the dissolved hydrohalic acid and volatile methylamine.^[^^95^^]^ The insoluble lead halide remains, as is consistent with experimental observations.

Additionally, hybrid perovskites decompose when heated to relatively low temperatures. While the standard annealing step at 100–150 °C improves crystallinity and preferential orientation, annealing at 200 °C even in vacuum, where no reactive oxygen or water is present, thermally decomposes the films: the PbI_2_ remains on the surface, while the methylammonium halide volatilizes.^[^^48^^]^ In situ XRD measurements taken during annealing of CH_3_NH_3_PbI_3−*x*_Cl_*x*_ films suggest that decomposition to PbI_2_ can occur after annealing 50 min at 100 °C in a nitrogen atmosphere and after only 10 min in air.^[^^136^^]^

Finally, an open question remains if the operation of the photovoltaic device inherently degrades the perovskite or another material in the stack even when isolated from the environment. Encapsulated TiO_2_ appears to generate deep electron traps under UV illumination, which degrade performance. More stable operation has been achieved using Al_2_O_3_ as the perovskite's scaffold, although the efficiency stabilized to about half of its initial value with most of the loss arising in the *V*_oc_ [Fig. 7(b)].^[^^134^^]^ Improvements in stability have also been reported in devices that do not use a hole-transport layer. By adding 5-ammoniumvaleric acid to the perovskite, using a combined scaffold made of TiO_2_ and ZrO_2_, and removing the hole-transport layer, stable operation at 10.5% efficiency was maintained for over 1000 h [Fig. 7(c)].^[^^135^^]^ Clearly as more materials are investigated for their contacting properties, it will become essential that they be simultaneously evaluated for their stability. Much work still remains in evaluating the stability of hybrid perovskites and their devices, discerning the mechanisms of instability, and developing new materials to overcome these challenges.

### Achieving high *V*_oc_ in perovskites with larger band gaps

CH_3_NH_3_PbBr_3_ has an estimated band gap (*E*_g_) of at least 2.2 eV, making it an attractive option for producing the top cell in a tandem solar cell. The highest *V*_oc_ produced from this material, however, is only 1.51 V,^[^^34^^]^ which yields a loss-in-potential of 0.7 V. In contrast, CH_3_NH_3_PbI_3_ loses only 0.5 V between its band gap (*E*_g_ = 1.6 eV) and *qV*_oc_, demonstrating that the iodide perovskite is much closer than the bromide perovskite to achieving its maximum efficiency. In comparison, GaInP (*E*_g_ ~ 1.9 eV), which is used for the top cell in high-performance tandem devices, has a loss-in-potential of ~0.5.^[^^150^^]^

The origin of the higher loss-in-potential of CH_3_NH_3_PbBr_3_ remains unclear. One possibility is reduced shunt resistance resulting from large pinholes in the CH_3_NH_3_PbBr_3_ film, since some increase in the *V*_oc_ has been seen for denser films.^[^^34^^]^ Another possibility is a larger density of charge traps or sub-band-gap states in the grain boundaries of CH_3_NH_3_PbBr_3_ than in CH_3_NH_3_PbI_3_. The poorer performance could also result from contacts with improper band alignment or inadequate interfacial passivation because the most recent record *V*_oc_ for this material was obtained simply by tuning the hole-transport layer.^[^^34^^,^^125^^]^

### Lead-free perovskites

The ultimate goal of materials used in optoelectronic applications should be based on the “triple-E” terms: energy efficient, economically inexpensive, and environmentally benign. The Pb-based halide perovskites have exhibited exceptional performance and will likely satisfy the first two criteria within the coming years; however, the presence of Pb is undesirable because of its toxicity. For a simple substitution, candidates should have a 2^+^ oxidation state to take the B site within the perovskite AB*X*_3_. These elements include those in the same group with Pb, such as Ge and Sn, but unfortunately their stability as B^2+^ is relatively low as compared with their stability in the B^4+^ state. CH_3_NH_3_SnI_3_ has been demonstrated to give a device efficiency as high as 6.4%, but it is much less stable against oxidation than CH_3_NH_3_PbI_3_.^[^^151^^,^^152^^]^ Other options can be found in the first row of the transition metals, such as Mn^2+^, Ni^2+^, Cu^2+^, and Zn^2+^, because their 2^+^ state is relatively stable as compared with their higher oxidation states, if any exist. In the case of copper, however, the pronounced Jahn–Teller distortion is likely to affect the band gap and crystal symmetry of the perovskite. Work on two-dimensional (2D) perovskites (A_2_B*X*_4_) has demonstrated the possibility of using transition metals in hybrid perovskites,^[^^154^^]^ which can then be adapted to 3D perovskites.^[^^153^^,^^154^^]^ In addition to non-toxicity, the criteria for selecting suitable replacements should also include the earth abundance^[^^155^^]^ and criticality, which accounts for the potential for a disruption in supply and how well such a disruption can be accommodated by the industry.^[^^156^^,^^157^^]^ The transition metals above are abundant non-critical materials.

## Conclusions and outlook

In just a few years, hybrid perovskites have already shown tremendous potential for fascinating fundamental and applied research. They exhibit electrical and optical properties that compare favorably with polycrystalline semiconductors, yet they are produced via solution-based processing. Material scientists wonder why—and wonder if they can make other materials behave similarly. Although much progress has been made in exploring the basic properties of CH_3_NH_3_PbI_3_ and CH_3_NH_3_PbI_3−*x*_Cl_*x*_, there are still many unanswered questions regarding the role of Cl^−^ during the synthesis and afterward, the origin of the hysteresis in electrical measurements, the optical properties, and the reason for the excellent performance in photovoltaics, particularly the long radiative lifetime. The challenge of determining the relationships between synthesis, structure, and function is a rich area for materials scientists.

Beyond the lead-iodide-based perovskites are countless unexplored avenues for the development of new materials. The tunability of the hybrid perovskites through their halides and organic and metal cations has many possibilities for systematically determining structure-function relationships to help design new perovskites. Part of these synthetic explorations should also include attention to the mechanisms of nucleation and growth of these crystals. A wide variety of morphologies have already been seen simply by changing the crystallization parameters, and achieving uniform nucleation and dense films, particularly on smooth substrates, remains challenging. As these films are produced at low temperature and often by rapid crystallization, it is likely that kinetics plays a significant role in the process.

Additionally, very little is known about these materials on the nanoscale, and it is likely that local variations in structure and composition influence the performance of devices. Already an example of this has been seen in the role of Cl^−^ in CH_3_NH_3_PbI_3−*x*_Cl_*x*_. As the perovskite mixtures become more complex to achieve new properties and to fine-tune electrical and optical characteristics, chemical analysis with high spatial resolution and sensitivity will become essential.

Because complex devices such as solar cells and lasers are not made from only a single material, there are also extensive opportunities for studying how perovskites form interfaces with other materials. Much of this work so far has centered around TiO_2_, but similar studies combining structural, electrical, optical, and theoretical techniques must examine other transport layers or scaffolds such as metal oxides and sulfides that will find application in future devices.

Lastly, for perovskites to have an impact beyond the laboratory, questions regarding the stability of the material and its device structures must be addressed as soon as possible. While concerns are real, similar stability issues were discovered and resolved previously in other technologies, for example in organic light-emitting diodes.^[^^158^^]^ Researchers can use the insights gained from those fields to speed their progress.

Around the world, chemists, physicists, and materials scientists are joining in the effort to realize the full potential of these versatile materials. It is a time for exploration and excitement as new discoveries are around every corner, and each one deepens our understanding of this new world of hybrid perovskites.

## References

[1] M.A. Green, K. Emery, Y. Hishikawa, W. Warta, and E.D. Dunlop: Solar cell efficiency tables (version 45). Prog. Photovolt. Res. Appl. 23, 1 (2015).

[2] A. Kojima, K. Teshima, Y. Shirai, and T. Miyasaka: Organometal halide perovskites as visible-light sensitizers for photovoltaic cells. J. Am. Chem. Soc. 131, 6050 (2009). 19366264 10.1021/ja809598r

[3] H.-S. Kim, C.-R. Lee, J.-H. Im, K.-B. Lee, T. Moehl, A. Marchioro, S.-J. Moon, R. Humphry-Baker, J.-H. Yum, J.E. Moser, M. Grätzel, and N.-G. Park: Lead iodide perovskite sensitized all-solid-state submicron thin film mesoscopic solar cell with efficiency exceeding 9%. Sci. Rep. 2, 591 (2012). 22912919 10.1038/srep00591 PMC3423636

[4] M. Liu, M.B. Johnston, and H.J. Snaith: Efficient planar heterojunction perovskite solar cells by vapour deposition. Nature 501, 395 (2013). 24025775 10.1038/nature12509

[5] J. Luo, J.-H. Im, M.T. Mayer, M. Schreier, M.K. Nazeeruddin, N.-G. Park, S.D. Tilley, H.J. Fan, and M. Gratzel: Water photolysis at 12.3% efficiency via perovskite photovoltaics and earth-abundant catalysts. Science 345, 1593 (2014). 25258076 10.1126/science.1258307

[6] Z.-K. Tan, R.S. Moghaddam, M.L. Lai, P. Docampo, R. Higler, F. Deschler, M. Price, A. Sadhanala, L.M. Pazos, D. Credgington, F. Hanusch, T. Bein, H.J. Snaith, and R.H. Friend: Bright light-emitting diodes based on organometal halide perovskite. Nat. Nanotechnol. 9, 687 (2014). 25086602 10.1038/nnano.2014.149

[7] Y.-H. Kim, H. Cho, J.H. Heo, T.-S. Kim, N. Myoung, C.-L. Lee, S.H. Im, and T.-W. Lee: Multicolored organic/inorganic hybrid perovskite light-emitting diodes. Adv. Mater. 27, 1248 (2014). 25420784 10.1002/adma.201403751

[8] F. Deschler, M. Price, S. Pathak, L.E. Klintberg, D. Jarausch, R. Higler, S. Hu, T. Leijtens, S.D. Stranks, H.J. Snaith, M. Atatu, R.T. Phillips, and R.H. Friend: High photoluminescence efficiency and optically pumped lasing in solution-processed mixed halide perovskite semiconductors. J. Phys. Chem. Lett. 5, 1421 (2014). 26269988 10.1021/jz5005285

[9] B.R. Sutherland, S. Hoogland, M.M. Adachi, C.T.O. Wong, E.H. Sargent, and S.E.T. Al: Conformal organohalide perovskites enable lasing on spherical resonators. ACS Nano 8, 10947 (2014). 25313937 10.1021/nn504856g

[10] Q. Zhang, S.T. Ha, X. Liu, T.C. Sum, and Q. Xiong: Room-temperature near-infrared high-q perovskite whispering-gallery planar nanolasers. Nano Lett. 14, 5995 (2014). 25118830 10.1021/nl503057g

[11] Y. Lee, J. Kwon, E. Hwang, C.-H. Ra, W.J. Yoo, J.-H. Ahn, J.H. Park, and J.H. Cho: High-performance perovskite-graphene hybrid photodetector. Adv. Mater. 27, 41 (2014). 25327379 10.1002/adma.201402271

[12] L. Dou, Y.M. Yang, J. You, Z. Hong, W.-H. Chang, G. Li, and Y. Yang: Solution-processed hybrid perovskite photodetectors with high detectivity. Nat. Commun. 5, 5404 (2014). 25410021 10.1038/ncomms6404

[13] M.A. Green, A. Ho-Baillie, and H.J. Snaith: The emergence of perovskite solar cells. Nat. Photon. 8, 506 (2014).

[14] T.C. Sum and N. Mathews: Advancements in perovskite solar cells: photophysics behind the photovoltaics. Energy Environ. Sci. 7, 2518 (2014).

[15] S. Kazim, M.K. Nazeeruddin, M. Grätzel, and S. Ahmad: Perovskite as light harvester: a game changer in photovoltaics. Angew. Chem. – Int. Ed. Engl. 53, 2812 (2014). 24519832 10.1002/anie.201308719

[16] H.S. Jung and N.-G. Park: Perovskite solar cells: from materials to devices. Small 11, 10 (2014). 25358818 10.1002/smll.201402767

[17] C. Li, X. Lu, W. Ding, L. Feng, Y. Gao, and Z. Guo: Formability of ABX_3_ (X = F, Cl, Br, I) halide perovskites. Acta Crystallogr. B 64, 702 (2008). 19029699 10.1107/S0108768108032734

[18] I. Swainson, L. Chi, J.H. Her, L. Cranswick, P. Stephens, B. Winkler, D.J. Wilson, and V. Milman: Orientational ordering, tilting and lone-pair activity in the perovskite methylammonium tin bromide, CH_3_NH_3_SnBr_3_. Acta Crystallogr. B 66, 422 (2010). 20631424 10.1107/S0108768110014734

[19] N. Onoda-Yamamuro, T. Matsuo, and H. Suga: Study of CH_3_NH_3_PbX_3_ (X = Cl, Br, I). J. Phys. Chem. Solids 53, 935 (1992).

[20] O. Knop, E. Wasylishen, M.A. White, T. Stanley, and J.M. Michiel: Alkylammonium lead halides. Part 2. CH3NH3PbX3 (X = C1, Br, I) perovskites: cuboctahedral halide cages with isotropic cation reorientation. Can. J. Chem. 68, 412 (1989).

[21] A. Poglitsch and D. Weber: Dynamic disorder in methylammoniumtrihalogenoplumbates (II) observed by millimeter-wave spectroscopy. J. Chem. Phys. 87, 6373 (1987).

[22] S.T. Williams, F. Zuo, C. Chueh, C. Liao, P. Liang, and A.K. Jen: Role of chloride in the morphological evolution of organo-lead halide perovskite thin films. ACS Nano 8, 10640 (2014). 25299303 10.1021/nn5041922

[23] D. Nanova, A.K. Kast, M. Pfannmo, C. Mu, L. Veith, I. Wacker, M. Agari, W. Hermes, P. Erk, W. Kowalsky, R.R.S. Der, and R. Lovrinc: Unraveling the nanoscale morphologies of mesoporous perovskite solar cells and their correlation to device performance. Nano Lett. 14, 2735 (2014). 24702643 10.1021/nl5006838

[24] C.C. Stoumpos, C.D. Malliakas, and M.G. Kanatzidis: Semiconducting tin and lead iodide perovskites with organic cations: phase transitions, high mobilities, and near-infrared photoluminescent properties. Inorg. Chem. 52, 9019 (2013). 23834108 10.1021/ic401215x

[25] B.R. Sutherland, S. Hoogland, M.M. Adachi, P. Kanjanaboos, C.T.O. Wong, J.J. McDowell, J. Xu, O. Voznyy, Z. Ning, A.J. Houtepen, and E.H. Sargent: Perovskite thin films via atomic layer deposition. Adv. Mater. 27, 53 (2014). 25359103 10.1002/adma.201403965

[26] I. Wharf, T. Gramstad, R. Makhija, and M. Onyszchuk: Synthesis and vibrational spectra of some lead(II) halide adducts with. Can. J. Chem. 54, 3430 (1976).

[27] R.J. Alvarado, J.M. Rosenberg, A. Andreu, J.C. Bryan, W.-Z. Chen, T. Ren, and K. Kavallieratos: Structural insights into the coordination and extraction of Pb(II) by disulfonamide ligands derived from o-phenylenediamine. Inorg. Chem. 44, 7951 (2005). 16241145 10.1021/ic051103r

[28] I. Persson, K. Lyczko, D. Lundberg, L. Eriksson, and A. Płaczek: Coordination chemistry study of hydrated and solvated lead(II) ions in solution and solid state. Inorg. Chem. 50, 1058 (2011). 21226482 10.1021/ic1017714

[29] G.P. Haight and J.R. Peterson: Chloro complexes of lead(II). Inorg. Chem. 4, 1073 (1965).

[30] H.L. Clever and F.J. Johnston: The solubility of some sparingly soluble lead salts: an evaluation of the solubility in water and aqueous electrolyte solution. J. Phys. Chem. Ref. Data 9, 751 (1980).

[31] D.B. Mitzi: Solution-processed inorganic semiconductors. J. Mater. Chem. 14, 2355 (2004).

[32] G.E. Eperon, V.M. Burlakov, P. Docampo, A. Goriely, and H.J. Snaith: Morphological control for high performance, solution-processed planar heterojunction perovskite solar cells. Adv. Funct. Mater. 24, 151 (2014).

[33] N.J. Jeon, J.H. Noh, Y.C. Kim, W.S. Yang, S. Ryu, and S. Il Seok: Solvent engineering for high-performance inorganic-organic hybrid perovskite solar cells. Nat. Mater. 13, 897 (2014). 24997740 10.1038/nmat4014

[34] J.H. Heo, D.H. Song, and S.H. Im: Planar CH_3_NH_3_PbBr_3_ hybrid solar cells with 10.4% power conversion efficiency, fabricated by controlled crystallization in the spin-coating process. Adv. Mater. 26, 8179 (2014). 25348285 10.1002/adma.201403140

[35] Z. Xiao, Q. Dong, C. Bi, Y. Shao, Y. Yuan, and J. Huang: Solvent annealing of perovskite-induced crystal growth for photovoltaic-device efficiency enhancement. Adv. Mater. 26, 6503 (2014). 25158905 10.1002/adma.201401685

[36] J.-H. Im, I.-H. Jang, N. Pellet, M. Grätzel, and N.-G. Park: Growth of CH_3_NH_3_PbI_3_ cuboids with controlled size for high-efficiency perovskite solar cells. Nat. Nanotechnol. 9, 927 (2014). 25173829 10.1038/nnano.2014.181

[37] G.E. Eperon, S.D. Stranks, C. Menelaou, M.B. Johnston, L.M. Herz, and H.J. Snaith: Formamidinium lead trihalide: a broadly tunable perovskite for efficient planar heterojunction solar cells. Energy Environ. Sci. 7, 982 (2014).

[38] J.H. Noh, S.H. Im, J.H. Heo, T.N. Mandal, and S. Il Seok: Chemical management for colorful, efficient, and stable inorganic–organic hybrid nanostructured solar cells. Nano Lett. 13, 1764 (2013). 23517331 10.1021/nl400349b

[39] G. Xing, N. Mathews, S.S. Lim, N. Yantara, X. Liu, D. Sabba, M. Grätzel, S. Mhaisalkar, and T.C. Sum: Low-temperature solution-processed wavelength-tunable perovskites for lasing. Nat. Mater. 13, 476 (2014). 24633346 10.1038/nmat3911

[40] A. Sadhanala, F. Deschler, T.H. Thomas, E. Dutton, K.C. Goedel, F.C. Hanusch, M.L. Lai, U. Steiner, T. Bein, P. Docampo, D. Cahen, and R.H. Friend: Preparation of single-phase films of CH_3_NH_3_Pb(I_1−x_Br_x_)_3_ with sharp optical band edges. J. Phys. Chem. Lett. 5, 2501 (2014). 26277936 10.1021/jz501332v

[41] N. Kitazawa, Y. Watanabe, and Y. Nakamura: Optical properties of CH_3_NH_3_PbX_3_ (X = halogen) and their mixed-halide crystals. J. Mater. Sci. Soc. 7, 3585 (2002).

[42] J. Burschka, N. Pellet, S.-J. Moon, R. Humphry-Baker, P. Gao, M.K. Nazeeruddin, and M. Grätzel: Sequential deposition as a route to high-performance perovskite-sensitized solar cells. Nature 499, 316 (2013). 23842493 10.1038/nature12340

[43] P. Docampo, F. Hanusch, S.D. Stranks, M. Döblinger, J.M. Feckl, M. Ehrensperger, N.K. Minar, M.B. Johnston, H.J. Snaith, and T. Bein: Solution deposition-conversion for planar heterojunction mixed halide perovskite solar cells. Adv. Energy Mater. 4, 1400355 (2014).

[44] Y. Kutes, L. Ye, Y. Zhou, S. Pang, B.D. Huey, and N.P. Padture: Direct observation of ferroelectric domains in solution-processed CH_3_NH_3_PbI_3_ perovskite thin films. J. Phys. Chem. Lett. 5, 3335 (2014). 26278441 10.1021/jz501697b

[45] R.G. Pearson: Hard and soft acids and bases, HSAB, part I. J. Chem. Educ. 45, 581 (1968).

[46] R.G. Pearson: Hard and soft acids and bases, HSAB, part II. J. Chem. Educ. 45, 643 (1968).

[47] L. Shimoni-livny, J.P. Glusker, and C.W. Bock: Lone pair functionality in divalent lead compounds. Inorg. Chem. 37, 1853 (1998).

[48] P. Pistor, J. Borchert, W. Fra, R. Csuk, and R. Scheer: Monitoring the phase formation of coevaporated lead halide perovskite thin films by in situ x-ray diffraction. J. Phys. Chem. Lett. 5, 3308 (2014). 26278437 10.1021/jz5017312

[49] Q. Chen, H. Zhou, Z. Hong, S. Luo, H. Duan, H. Wang, Y. Liu, G. Li, and Y. Yang: Planar heterojunction perovskite solar cells via vapor-assisted solution process. J. Am. Chem. Soc. 136, 622 (2014). 24359486 10.1021/ja411509g

[50] S.T. Ha, X. Liu, Q. Zhang, D. Giovanni, T.C. Sum, and Q. Xiong: Synthesis of organic-inorganic lead halide perovskite nanoplatelets: towards high-performance perovskite solar cells and optoelectronic devices. Adv. Opt. Mater. 2, 838 (2014).

[51] M.M. Lee, J. Teuscher, T. Miyasaka, T.N. Murakami, and H.J. Snaith: Efficient hybrid solar cells based on meso-superstructured organometal halide perovskites. Science 338, 643 (2012). 23042296 10.1126/science.1228604

[52] G. Grancini, S. Marras, M. Prato, C. Giannini, C. Quarti, F. De Angelis, M. De Bastiani, G.E. Eperon, H.J. Snaith, L. Manna, and A. Petrozza: The impact of the crystallization processes on the structural and optical properties of hybrid perovskite films for photovoltaics. J. Phys. Chem. Lett. 5, 3836 (2014). 26278757 10.1021/jz501877h

[53] V. D'Innocenzo, G. Grancini, M.J.P. Alcocer, A.R.S. Kandada, S.D. Stranks, M.M. Lee, G. Lanzani, H.J. Snaith, and A. Petrozza: Excitons versus free charges in organo-lead tri-halide perovskites. Nat. Commun. 5, 3586 (2014). 24710005 10.1038/ncomms4586

[54] S. Colella, E. Mosconi, P. Fedeli, A. Listorti, F. Gazza, F. Orlandi, P. Ferro, T. Besagni, A. Rizzo, G. Calestani, G. Gigli, F. De Angelis, and R. Mosca: MAPbI_3−x_Cl_x_ mixed halide perovskite for hybrid solar cells: the role of chloride as dopant on the transport and structural properties. Chem. Mater. 25, 4613 (2013).

[55] C. Wehrenfennig, G.E. Eperon, M.B. Johnston, H.J. Snaith, and L.M. Herz: High charge carrier mobilities and lifetimes in organolead trihalide perovskites. Adv. Mater. 26, 1584 (2014). 24757716 10.1002/adma.201305172 PMC4722848

[56] S.D. Stranks, G.E. Eperon, G. Grancini, C. Menelaou, M.J.P. Alcocer, T. Leijtens, L.M. Herz, A. Petrozza, and H.J. Snaith: Electron-hole diffusion lengths exceeding 1 micrometer in an organometal trihalide perovskite absorber. Science 342, 341 (2013). 24136964 10.1126/science.1243982

[57] G. Xing, N. Mathews, S. Sun, S.S. Lim, Y.M. Lam, M. Grätzel, S. Mhaisalkar, and T.C. Sum: Long-range balanced electron- and hole-transport lengths in organic-inorganic CH_3_NH_3_PbI_3_. Science 342, 344 (2013). 24136965 10.1126/science.1243167

[58] Y. Zhao and K. Zhu: CH_3_NH_3_Cl-assisted one-step solution growth of CH_3_NH_3_PbI_3_: structure , charge-carrier dynamics, and photovoltaic properties of perovskite solar cells. J. Phys. Chem. C 118, 9412 (2014).

[59] A. Romeo, M. Terheggen, D. Abou-Ras, D.L. Bätzner, F.-J. Haug, M. Kälin, D. Rudmann, and A.N. Tiwari: Development of thin-film Cu(In,Ga)Se_2_ and CdTe solar cells. Prog. Photovolt. Res. Appl. 12, 93 (2004).

[60] Y. Tidhar, E. Edri, H. Weissman, D. Zohar, G. Hodes, D. Cahen, B. Rybtchinski, and S. Kirmayer: Crystallization of methyl ammonium lead halide perovskites: implications for photovoltaic applications. J. Am. Chem. Soc. 136, 13249 (2014). 25171634 10.1021/ja505556s

[61] H. Yu, F. Wang, F. Xie, W. Li, J. Chen, and N. Zhao: The role of chlorine in the formation process of “CH_3_NH_3_PbI_3−x_Cl_x_” perovskite. Adv. Funct. Mater. 24, 7102 (2014).

[62] A. Buin, P. Pietsch, J. Xu, O. Voznyy, A.H. Ip, R. Comin, and E.H. Sargent: Materials processing routes to trap-free halide perovskites. Nano Lett. 14, 6281 (2014). 25296282 10.1021/nl502612m

[63] T. Baikie, Y. Fang, J.M. Kadro, M. Schreyer, F. Wei, S.G. Mhaisalkar, M. Graetzel, and T.J. White: Synthesis and crystal chemistry of the hybrid perovskite (CH_3_NH_3_)PbI_3_ for solid-state sensitised solar cell applications. J. Mater. Chem. A 1, 5628 (2013).

[64] J. Even, L. Pedesseau, J.-M. Jancu, and C. Katan: Importance of spin–orbit coupling in hybrid organic/inorganic perovskites for photovoltaic applications. J. Phys. Chem. Lett. 4, 2999 (2013).

[65] L. Huang and W.R.L. Lambrecht: Electronic band structure, phonons, and exciton binding energies of halide perovskites CsSnCl_3_, CsSnBr_3_, and CsSnI_3_. Phys. Rev. B 88, 165203 (2013).

[66] G. Giorgi, J. Fujisawa, H. Segawa, and K. Yamashita: Small photocarrier effective masses featuring ambipolar transport in methylammonium lead iodide perovskite: a density functional analysis. J. Phys. Chem. Lett. 4, 4213 (2013). 26296167 10.1021/jz4023865

[67] F. Brivio, A.B. Walker, and A. Walsh: Structural and electronic properties of hybrid perovskites for high-efficiency thin-film photovoltaics from first-principles. APL Mater. 1, 042111 (2013).

[68] Q. Wang, Y. Shao, H. Xie, L. Lyu, X. Liu, Y. Gao, and J. Huang: Qualifying composition dependent p and n self-doping in CH_3_NH_3_PbI_3_. Appl. Phys. Lett. 105, 163508 (2014).

[69] Y. Takahashi, R. Obara, Z.-Z. Lin, Y. Takahashi, T. Naito, T. Inabe, S. Ishibashi, and K. Terakura: Charge-transport in tin-iodide perovskite CH_3_NH_3_SnI_3_: origin of high conductivity. Dalton Trans. 40, 5563 (2011). 21494720 10.1039/c0dt01601b

[70] D. Venkateshvaran, M. Nikolka, A. Sadhanala, V. Lemaur, M. Zelazny, M. Kepa, M. Hurhangee, A.J. Kronemeijer, V. Pecunia, I. Nasrallah, I. Romanov, K. Broch, I. McCulloch, D. Emin, Y. Olivier, J. Cornil, D. Beljonne, and H. Sirringhaus: Approaching disorder-free transport in high-mobility conjugated polymers. Nature 515, 384 (2014). 25383522 10.1038/nature13854

[71] J. You, L. Dou, Z. Hong, G. Li, and Y. Yang: Recent trends in polymer tandem solar cells research. Prog. Polym. Sci. 38, 1909 (2013).

[72] C.S.S. Sandeep, S. Cate, J.M. Schins, T.J. Savenije, Y. Liu, M. Law, S. Kinge, A.J. Houtepen, and L.D.A. Siebbeles: High charge-carrier mobility enables exploitation of carrier multiplication in quantum-dot films. Nat. Commun. 4, 2360 (2013). 23974282 10.1038/ncomms3360 PMC3759061

[73] Q. Long, S.A. Dinca, E.A. Schiff, M. Yu, and J. Theil: Electron and hole drift mobility measurements on thin film CdTe solar cells. Appl. Phys. Lett. 105, 042106 (2014).

[74] B. Shin, O. Gunawan, Y. Zhu, N.A. Bojarczuk, S.J. Chey, and S. Guha: Thin film solar cell with 8.4% power conversion efficiency using an earth-abundant Cu_2_ZnSnS_4_ absorber. Prog. Photovolt. Res. Appl. 21, 72 (2013).

[75] G. Brown, V. Faifer, A. Pudov, S. Anikeev, E. Bykov, M. Contreras, and J. Wu: Determination of the minority carrier diffusion length in compositionally graded Cu(In,Ga)Se_2_ solar cells using electron beam induced current. Appl. Phys. Lett. 96, 022104 (2010).

[76] T.I. Kamins: Hall mobility in chemically deposited polycrystalline silicon. J. Appl. Phys. 42, 43574365 (1971).

[77] M.A. Green: Silicon Solar Cells: Advanced Principles & Practice (University of New South Wales, Sydney, NSW., 1995), p. 76.

[78] J.S. Blakemore: Semiconducting and other major properties of gallium arsenide. J. Appl. Phys. 53, R123 (1982).

[79] T. Leijtens, S.D. Stranks, G.E. Eperon, R. Lindblad, E.M.J. Johansson, J.M. Ball, M.M. Lee, H.J. Snaith, and I.J. McPherson: Electronic properties of meso-superstructured and planar organometal halide perovskite films: charge trapping, photodoping, and carrier mobility. ACS Nano 8, 7147 (2014). 24949826 10.1021/nn502115k

[80] Q. Chen, H. Zhou, T.-B. Song, S. Luo, Z. Hong, H.-S. Duan, L. Dou, Y. Liu, and Y. Yang: Controllable self-induced passivation of hybrid lead iodide perovskites toward high performance solar cells. Nano Lett. 14, 4158 (2014). 24960309 10.1021/nl501838y

[81] A. Dualeh, T. Moehl, N. Tétreault, J. Teuscher, P. Gao, M.K. Nazeeruddin, and M. Grätzel: Impedance spectroscopic analysis of lead iodide perovskite-sensitized solid-state solar cells. ACS Nano 8, 362 (2014). 24341597 10.1021/nn404323g

[82] V. Gonzalez-Pedro, E.J. Juarez-Perez, W.-S. Arsyad, E.M. Barea, F. Fabregat-santiago, I. Mora-Sero, and J. Bisquert: General working principles of CH_3_NH_3_PbX_3_ perovskite solar cells. Nano Lett. 14, 888 (2014). 24397375 10.1021/nl404252e

[83] E. Edri, S. Kirmayer, S. Mukhopadhyay, K. Gartsman, G. Hodes, and D. Cahen: Elucidating the charge carrier separation and working mechanism of CH_3_NH_3_PbI_(3−x)_Cl_(x)_ perovskite solar cells. Nat. Commun. 5, 3461 (2014). 24613942 10.1038/ncomms4461

[84] E. Edri, S. Kirmayer, A. Henning, S. Mukhopadhyay, K. Gartsman, Y. Rosenwaks, G. Hodes, and D. Cahen: Why lead methylammonium tri-iodide perovskite-based solar cells require a mesoporous electron transporting scaffold (but not necessarily a hole conductor). Nano Lett. 14, 1000 (2014). 24475878 10.1021/nl404454h

[85] H.-S. Kim, I. Mora-Sero, V. Gonzalez-Pedro, F. Fabregat-Santiago, E.J. Juarez-Perez, N.-G. Park, and J. Bisquert: Mechanism of carrier accumulation in perovskite thin-absorber solar cells. Nat. Commun. 4, 2242 (2013). 23900067 10.1038/ncomms3242

[86] V.W. Bergmann, S.A.L. Weber, F.J. Ramos, M.K. Nazeeruddin, M. Grätzel, D. Li, A.L. Domanski, I. Lieberwirth, S. Ahmad, and R. Berger: Real-space observation of unbalanced charge distribution inside a perovskite-sensitized solar cell. Nat. Commun. 5, 5001 (2014). 25242041 10.1038/ncomms6001

[87] L. Tarricone, N. Romeo, G. Sberveglier, and S. Mora: Electron and hole diffusion length investigation in CdTe thin films by SPV method. Sol. Energy Mater. 7, 343 (1982).

[88] O.V. Mikhnenko, H. Azimi, M. Scharber, M. Morana, P.W.M. Blom, and M.A. Loi: Exciton diffusion length in narrow bandgap polymers. Energy Environ. Sci. 5, 6960 (2012).

[89] G.I. Koleilat, L. Levina, H. Shukla, S.H. Myrskog, S. Hinds, A.G. Pattantyus-abraham, and E.H. Sargent: Efficient, stable infrared photovoltaics based on solution-cast colloidal quantum dots. ACS Nano 2, 833 (2008). 19206479 10.1021/nn800093v

[90] R. Gottesman, E. Haltzi, L. Gouda, S. Tirosh, Y. Bouhadana, A. Zaban, E. Mosconi, and F. De Angelis: Extremely slow photoconductivity response of CH_3_NH_3_PbI_3_ perovskites suggesting structural changes under working conditions. J. Phys. Chem. Lett. 5, 2662 (2014). 26277960 10.1021/jz501373f

[91] H.J. Snaith, A. Abate, J.M. Ball, G.E. Eperon, T. Leijtens, N.K. Noel, S.D. Stranks, J.T. Wang, K. Wojciechowski, and W. Zhang: Anomalous hysteresis in perovskite solar sells. J. Phys. Chem. Lett. 5, 1511 (2014). 26270088 10.1021/jz500113x

[92] E.L. Unger, E.T. Hoke, C.D. Bailie, W.H. Nguyen, A.R. Bowring, T. Heumuller, M.G. Christoforo, and M.D. McGehee: Hysteresis and transient behavior in current-voltage measurements of hybrid-perovskite absorber solar cells. Energy Environ. Sci. 7, 3690 (2014).

[93] R.S. Sanchez, V. Gonzalez-Pedro, J.-W. Lee, N.-G. Park, Y.S. Kang, I. Mora-Sero, and J. Bisquert: Slow dynamic processes in lead halide perovskite solar cells. Characteristic times and hysteresis. J. Phys. Chem. Lett. 5, 2357 (2014). 26279559 10.1021/jz5011187

[94] B. Noheda, Z. Zhong, D. Cox, G. Shirane, S.-E. Park, and P. Rehrig: Electric-field-induced phase transitions in rhombohedral Pb(Zn_1/3_Nb_2/3_)_1−x_Ti_x_O_3_. Phys. Rev. B 65, 224101 (2002).

[95] J.M. Frost, K.T. Butler, F. Brivio, C.H. Hendon, M. van Schilfgaarde, and A. Walsh: Atomistic origins of high-performance in hybrid halide perovskite solar cells. Nano Lett. 14, 2584 (2014). 24684284 10.1021/nl500390f PMC4022647

[96] E.J. Juarez-Perez, R.S. Sanchez, L. Badia, G. Garcia-Belmonte, Y.S. Kang, I. Mora-Sero, and J. Bisquert: Photoinduced giant dielectric constant in lead halide perovskite solar cells. J. Phys. Chem. Lett. 5, 2390 (2014). 26279565 10.1021/jz5011169

[97] S.Y. Yang, J. Seidel, S.J. Byrnes, P. Shafer, C.-H. Yang, M.D. Rossell, P. Yu, Y.-H. Chu, J.F. Scott, J.W. Ager, L.W. Martin, and R. Ramesh: Above-bandgap voltages from ferroelectric photovoltaic devices. Nat. Nanotechnol. 5, 143 (2010). 20062051 10.1038/nnano.2009.451

[98] J. Seidel, D. Fu, S.-Y. Yang, E. Alarcón-Lladó, J. Wu, R. Ramesh, and J.W. Ager: Efficient photovoltaic current generation at ferroelectric domain walls. Phys. Rev. Lett. 107, 126805 (2011). 22026787 10.1103/PhysRevLett.107.126805

[99] P. Lunkenheimer, V. Bobnar, A.V. Pronin, A.I. Ritus, A.A. Volkov, and A. Loidl: Origin of apparent colossal dielectric constants. Phys. Rev. B 66, 052105 (2002).

[100] F.M. Pontes, E.R. Leite, E. Longo, J.A. Varela, E.B. Araujo, and J.A. Eiras: Effects of the postannealing atmosphere on the dielectric properties of (Ba, Sr)TiO_3_ capacitors: evidence of an interfacial space charge layer. Appl. Phys. Lett. 76, 2433 (2000).

[101] T.M. Shaw, S. Trolier-McKinstry, and P.C. McIntyre: The properties of ferroelectric films at small dimensions. Annu. Rev. Mater. Sci. 30, 263 (2000).

[102] J. Mizusaki, K. Arai, and K. Fueki: Ionic conduction of the perovskite-type halides. Solid State Ion. 11, 203 (1983).

[103] Z. Xiao, Y. Yuan, Y. Shao, Q. Wang, Q. Dong, C. Bi, P. Sharma, A. Gruverman, and J. Huang: Giant switchable photovoltaic effect in organometal trihalide perovskite devices. Nat. Mater. 1, 10 (2014). 10.1038/nmat4150 25485985

[104] M. Stuckelberger, B. Niesen, M. Filipic, S. Moon, J. Yum, M. Topic, and C. Ballif: Complex refractive index spectra of CH_3_NH_3_PbI_3_ perovskite thin films determined by spectroscopic ellipsometry and spectrophotometry. J. Phys. Chem. Lett. 6, 66 (2015). 26263093 10.1021/jz502471h

[105] Q. Lin, A. Armin, R.C.R. Nagiri, P.L. Burn, and P. Meredith: Electro-optics of perovskite solar cells. Nat. Photonics 9, 106 (2014).

[106] M. Saba, M. Cadelano, D. Marongiu, F. Chen, V. Sarritzu, N. Sestu, C. Figus, M. Aresti, R. Piras, A. Geddo Lehmann, C. Cannas, A. Musinu, F. Quochi, A. Mura, and G. Bongiovanni: Correlated electron-hole plasma in organometal perovskites. Nat. Commun. 5, 5049 (2014). 25266869 10.1038/ncomms6049

[107] K. Tanaka, T. Takahashi, T. Ban, T. Kondo, K. Uchida, and N. Miura: Comparative study on the excitons in lead-halide-based perovskite-type crystals CH_3_NH_3_PbBr_3_ CH_3_NH_3_PbI_3_. Solid State Commun. 127, 619 (2003).

[108] N.K. Noel, A. Abate, S.D. Stranks, E.S. Parrott, V.M. Burlakov, A. Goriely, H.J. Snaith, and N.E.T. Al: Enhanced photoluminescence and solar cell performance via lewis base passivation of organic-inorganic lead halide perovskites. ACS Nano 8, 9815 (2014). 25171692 10.1021/nn5036476

[109] S.D. Stranks, V.M. Burlakov, T. Leijtens, J.M. Ball, A. Goriely, and H.J. Snaith: Recombination kinetics in organic-inorganic perovskites: excitons, free charge, and subgap states. Phys. Rev. Appl. 2, 034007 (2014).

[110] C. Wehrenfennig, M. Liu, H.J. Snaith, M.B. Johnston, and L.M. Herz: Homogeneous emission line broadening in the organo lead halide perovskite CH_3_NH_3_PbI_3_. J. Phys. Chem. Lett. 5, 1300 (2014). 26269971 10.1021/jz500434p

[111] J.S. Manser and P.V. Kamat: Band filling with free charge carriers in organometal halide perovskites. Nat. Photonics 8, 737 (2014).

[112] N. Pellet, P. Gao, G. Gregori, T.-Y. Yang, M.K. Nazeeruddin, J. Maier, and M. Grätzel: Mixed-organic-cation perovskite photovoltaics for enhanced solar-light harvesting. Angew. Chem. – Int. Ed. Engl. 53, 3151 (2014). 24554633 10.1002/anie.201309361

[113] R.K. Ahrenkiel: Measurement of minority-carrier lifetime by time-resolved photoluminescence. Solid. State. Electron. 35, 239 (1992).

[114] A. Maalej, Y. Abid, A. Kallel, A. Daoud, A. Lautié, and F. Romain: Phase transitions and crystal dynamics in the cubic perovskite CH_3_NH_3_PBCl_3_. Solid State Commun. 103, 279 (1997).

[115] D.M. Calistru, L. Mihut, S. Lefrant, and I. Baltog: Identification of the symmetry of phonon modes in CsPbCl_3_ in phase IV by Raman and resonance-Raman scattering. J. Appl. Phys. 82, 5391 (1997).

[116] C. Quarti, G. Grancini, E. Mosconi, P. Bruno, J.M. Ball, M.M. Lee, H.J. Snaith, A. Petrozza, and F. De Angelis: The Raman spectrum of the CH_3_NH_3_PbI_3_ hybrid perovskite: interplay of theory and experiment. J. Phys. Chem. Lett. 5, 279 (2014). 26270700 10.1021/jz402589q

[117] N. Onoda-Yamamuro, T. Matsuo, and H. Suga: Calorimetric and IR spectroscopic studies of phase transitions in methylammonium trihalogenoplumbates (II). J. Phys. Chem. Solids 51, 1383 (1990).

[118] S. Hirotsu: Far-infrared reflectivity spectra of CsPbCI_3_. Phys. Lett. 41, 55 (1972).

[119] S. Ito, S. Tanaka, K. Manabe, and H. Nishino: Effects of surface blocking layer of Sb_2_S_3_ on nanocrystalline TiO_2_ for CH_3_NH_3_PbI_3_ perovskite solar cells. J. Phys. Chem. C 118, 16995 (2014).

[120] A. Abrusci, S.D. Stranks, P. Docampo, H.-L. Yip, A.K.-Y. Jen, and H.J. Snaith: High-performance perovskite-polymer hybrid solar cells via electronic coupling with fullerene monolayers. Nano Lett. 13, 3124 (2013). 23772773 10.1021/nl401044q

[121] J. You, Z. Hong, Y.M. Yang, Q. Chen, M. Cai, T-B. Song, C-C. Chen, S. Lu, Y. Liu, H. Zhou, and Y. Yang: Low-temperature solution-processed perovskite solar cells with high efficiency and flexibility. ACS Nano 8, 1674 (2014). 24386933 10.1021/nn406020d

[122] B. Xu, E. Sheibani, P. Liu, J. Zhang, H. Tian, N. Vlachopoulos, G. Boschloo, L. Kloo, A. Hagfeldt, and L. Sun: Carbazole-based hole-transport materials for efficient solid-state dye-sensitized solar cells and perovskite solar cells. Adv. Mater. 26, 6629 (2014). 25124337 10.1002/adma.201402415

[123] Z. Zhu, Y. Bai, H.K.H. Lee, C. Mu, T. Zhang, L. Zhang, J. Wang, H. Yan, S.K. So, and S. Yang: Polyfluorene derivatives are high-performance organic hole-transporting materials for inorganic−organic hybrid perovskite solar cells. Adv. Funct. Mater. 24, 7357 (2014).

[124] N.J. Jeon, J. Lee, J.H. Noh, M.K. Nazeeruddin, and S. Il Seok: Efficient inorganic−organic hybrid perovskite solar cells based on pyrene arylamine derivatives as hole-transporting materials. J. Am. Chem. Soc. 135, 19087 (2013). 24313292 10.1021/ja410659k

[125] S. Ryu, J.H. Noh, N.J. Jeon, Y. Chan Kim, W.S. Yang, J. Seo, and S. Il Seok: Voltage output of efficient perovskite solar cells with high open-circuit voltage and fill factor. Energy Environ. Sci. 3, 2614 (2014).

[126] M.P. De Jong, L.J. Van Ijzendoorn, and M.J.A. De Voigt: Stability of the interface between indium-tin-oxide and poly(3,4-ethylenedioxythiophene/poly(styrenesulfonate) in polymer light-emitting diodes. Appl. Phys. Lett. 77, 2255 (2000).

[127] E. Voroshazi, B. Verreet, A. Buri, R. Müller, D. Di Nuzzo, and P. Heremans: Influence of cathode oxidation via the hole extraction layer in polymer: fullerene solar cells. Org. Electron. 12, 736 (2011).

[128] S. Kim, A. Konar, W.-S. Hwang, J.H. Lee, J. Lee, J. Yang, C. Jung, H. Kim, J.-B. Yoo, J.-Y. Choi, Y.W. Jin, S.Y. Lee, D. Jena, W. Choi, and K. Kim: High-mobility and low-power thin-film transistors based on multilayer MoS_2_ crystals. Nat. Commun. 3, 1011 (2012). 22910357 10.1038/ncomms2018

[129] Z. Zhu, Y. Bai, T. Zhang, Z. Liu, X. Long, Z. Wei, Z. Wang, L. Zhang, J. Wang, F. Yan, and S. Yang: High-performance hole-extraction layer of sol-gel-processed NiO nanocrystals for inverted planar perovskite solar cells. Angew. Chem. – Int. Ed. Engl. 53, 12571 (2014). 25044246 10.1002/anie.201405176

[130] Y. Zhao, A.M. Nardes, and K. Zhu: Effective hole extraction using MoOx-Al contact in perovskite CH_3_NH_3_PbI_3_ solar cells. Appl. Phys. Lett. 104, 213906 (2014).

[131] V. Shrotriya, G. Li, Y. Yao, C.-W. Chu, and Y. Yang: Transition metal oxides as the buffer layer for polymer photovoltaic cells. Appl. Phys. Lett. 88, 073508 (2006).

[132] C. Tao, S. Ruan, G. Xie, X. Kong, L. Shen, F. Meng, C. Liu, X. Zhang, W. Dong, and W. Chen: Role of tungsten oxide in inverted polymer solar cells. Appl. Phys. Lett. 94, 043311 (2009).

[133] I. Chung, J. Song, J. Im, J. Androulakis, C.D. Malliakas, H. Li, A.J. Freeman, J.T. Kenney, and M.G. Kanatzidis: CsSnI_3_: semiconductor or metal? High electrical conductivity and strong near-infrared photoluminescence from a single material. High hole mobility and phase-transitions. J. Am. Chem. Soc. 3, 8579 (2012). 22578072 10.1021/ja301539s

[134] T. Leijtens, G.E. Eperon, S. Pathak, A. Abate, M.M. Lee, and H.J. Snaith: Overcoming ultraviolet light instability of sensitized TiO_2_ with meso-superstructured organometal tri-halide perovskite solar cells. Nat. Commun. 4, 2885 (2013). 24301460 10.1038/ncomms3885

[135] A. Mei, X. Li, L. Liu, Z. Ku, T. Liu, Y. Rong, M. Xu, M. Hu, J. Chen, Y. Yang, M. Gratzel, and H. Han: A hole-conductor-free, fully printable mesoscopic perovskite solar cell with high stability. Science 345, 295 (2014). 25035487 10.1126/science.1254763

[136] K.W. Tan, D.T. Moore, M. Saliba, H. Sai, L.A. Estroff, T. Hanrath, H.J. Snaith, and U. Wiesner: Thermally induced structural evolution and performance of mesoporous block copolymer-directed alumina perovskite solar cells. ACS Nano 8, 4730 (2014). 24684494 10.1021/nn500526t PMC4046796

[137] W. Shockley and H.J. Queisser: Detailed balance limit of efficiency of p-n junction solar cells. J. Appl. Phys. 32, 510 (1961).

[138] C.D. Bailie, M.G. Christoforo, J.P. Mailoa, A.R. Bowring, E.L. Unger, W.H. Nguyen, J. Burschka, N. Pellet, J.Z. Lee, M. Grätzel, R. Noufi, T. Buonassisi, A. Salleo, and M.D. McGehee: Polycrystalline tandem photovoltaics using perovskites on top of silicon and CIGS. Energy Environ. Sci. (2015). doi: 10.1039/c4ee03322a.

[139] T.P. White, N.N. Lal, and K.R. Catchpole: Tandem solar cells based on high-efficiency c-Si bottom cells: top cell requirements for >30 % efficiency. IEEE J. Photovolt. 4, 208 (2014).

[140] G.E. Eperon, V.M. Burlakov, A. Goriely, and H.J. Snaith: Neutral color semitransparent microstructured perovskite solar cells. ACS Nano 8, 591 (2014). 24467381 10.1021/nn4052309

[141] M.G. Kang, N. Park, and Y.J. Park: Manufacturing method for transparent electric windows using dye-sensitized TiO_2_ solar cells. Sol. Energy Mater. Sol. Cells 75, 475 (2003).

[142] R.R. Lunt and V. Bulovic: Transparent, near-infrared organic photovoltaic solar cells for window and energy-scavenging applications. Appl. Phys. Lett. 98, 113305 (2011).

[143] M.-G. Kang, T. Xu, H.J. Park, X. Luo, and L.J. Guo: Efficiency enhancement of organic solar cells using transparent plasmonic Ag nanowire electrodes. Adv. Mater. 22, 4378 (2010). 20734378 10.1002/adma.201001395

[144] M.W. Rowell, M.A. Topinka, M.D. McGehee, H-J. Prall, G. Dennler, N.S. Sariciftci, L. Hu, and G. Gruner: App. Phys. Lett. 88, 233506 (2006).

[145] X. Wang, L. Zhi, and K. Müllen: Transparent, conductive graphene electrodes for dye-sensitized solar cells. Nano Lett. 8, 323 (2008). 18069877 10.1021/nl072838r

[146] S. Höfle, A. Schienle, C. Bernhard, M. Bruns, U. Lemmer, and A. Colsmann: Solution processed, white emitting tandem organic light-emitting diodes with inverted device architecture. Adv. Mater. 26, 5155 (2014). 24899163 10.1002/adma.201400332

[147] S. Hu, C. Xiang, S. Haussener, A.D. Berger, and N.S. Lewis: An analysis of the optimal band gaps of light absorbers in integrated tandem photoelectrochemical water-splitting systems. Energy Environ. Sci. 6, 2984 (2013).

[148] O. Khaselev: A monolithic photovoltaic-photoelectrochemical device for hydrogen production via water splitting. Science 280, 425 (1998). 9545218 10.1126/science.280.5362.425

[149] S. Hu, M.R. Shaner, J.A. Beardslee, M. Lichterman, B.S. Brunschwig, and N.S. Lewis: Amorphous TiO_2_ coatings stabilize Si, GaAs, and GaP photoanodes for efficient water oxidation. Science 344, 2547 (2014). 10.1126/science.1251428 24876492

[150] T. Takamoto, E. Ikeda, H. Kurita, and M. Ohmori: Over 30% efficient InGaP/GaAs tandem solar cells. Appl. Phys. Lett. 70, 381 (1997).

[151] F. Hao, C.C. Stoumpos, D.H. Cao, R.P.H. Chang, and M.G. Kanatzidis: Lead-free solid-state organic–inorganic halide perovskite solar cells. Nat. Photonics 8, 489 (2014).

[152] N.K. Noel, S.D. Stranks, A. Abate, C. Wehrenfennig, S. Guarnera, A. Haghighirad, A. Sadhanala, G.E. Eperon, S.K. Pathak, M.B. Johnston, A. Petrozza, L. Herz, and H. Snaith: Lead-free organic-inorganic tin halide perovskites for photovoltaic applications. Energy Environ. Sci. 7, 3061 (2014).

[153] D.B. Mitzi: Templating and structural engineering in organic–inorganic perovskites. J. Chem. Soc. Dalt. Trans. 1, 1 (2001).

[154] G.S. Long, M. Wei, and R.D. Willett: Crystal structures and magnetic properties of a novel layer perovskite system: (3-Picoliniumylammonium)CuX_4_ (X = Cl, Br). Inorg. Chem. 36, 3102 (1997). 11669963 10.1021/ic960849+

[155] G.B. Haxel, J.B. Hedrick, and G.J. Orris: US Geological Survey fact sheet 087-02. US Geol. Surv. (2005). <http://pubs.usgs.gov/fs/2002/fs087-02/>.

[156] Report on critical raw materials for the EU: critical raw materials profiles. *Eur. Commun.* (2013). <http://ec.europa.eu/enterprise/policies/raw-materials/files/docs/crm-critical-material-profiles_en.pdf>.

[157] Critical materials strategy summary. US Department of Energy (2011). <http://energy.gov/node/349057>.

[158] F. So and D. Kondakov: Degradation mechanisms in small-molecule and polymer organic light-emitting diodes. Adv. Mater. 22, 3762 (2010). 20491088 10.1002/adma.200902624

